# Unveiling the Power of Computational Tools in Chiral Liquid Chromatography

**DOI:** 10.3390/molecules30153218

**Published:** 2025-07-31

**Authors:** Rita Lima, Rui P. P. Neves, Pedro A. Fernandes, Artur M. S. Silva, Carla Fernandes

**Affiliations:** 1 Laboratório de Química Orgânica e Farmacêutica, Departamento de Ciências Químicas, Faculdade de Farmácia, Universidade do Porto, Rua Jorge Viterbo Ferreira, 228, 4050-313 Porto, Portugal; ritaalexandralima@gmail.com; 2 Centro Interdisciplinar de Investigação Marinho e Ambiental (CIIMAR), Edifício do Terminal de Cruzeiros do Porto de Leixões, Av. General Norton de Matos, s/n, 4050-208 Matosinhos, Portugal; 3 LAQV, REQUIMTE, Departamento de Química e Bioquímica, Faculdade de Ciências, Universidade do Porto, Rua do Campo Alegre, s/n, 4169-007 Porto, Portugal; rui.neves@fc.up.pt (R.P.P.N.); pafernan@fc.up.pt (P.A.F.); 4 LAQV, REQUIMTE, Departamento de Química, Universidade de Aveiro, 3810-193 Aveiro, Portugal; artur.silva@ua.pt

**Keywords:** chiral liquid chromatography, chiral stationary phases, computational studies, enantioseparation, molecular modeling, molecular recognition

## Abstract

Chiral liquid chromatography (cLC) using chiral stationary phases (CSPs) has become a crucial technique for separating enantiomers. Understanding enantiomeric discrimination is essential for improving chromatographic conditions and elucidating chiral molecular recognition; the computational methods are extremely helpful for this. To assess the relevance of the association of these two approaches and to analyze the current trends, in this review, a systematic analysis of the scientific literature was performed, covering recently published works (from 2015 to January 2025) on enantioseparation by cLC using CSPs and computational studies. CSPs based on polysaccharides and Pirkle-type were the most described (accounting for 52% and 14% of the studies, respectively). Regarding the computational methods, molecular docking and molecular dynamics (MD) were the most reported (accounting for 50% and 25% of the studies, respectively). In the articles surveyed, a significant growth in research concerning both cLC enantioseparation and computational studies is evident, emphasizing the benefit of the synergy between these two approaches.

## 1. Introduction

Over the last few decades, chiral liquid chromatography (cLC) has proven to be one of the most versatile and widely applied techniques for the analysis and purification of enantiomers in diverse research fields, such as food [1,2], pharmaceutical [3], biomedical [4], environmental [5,6], forensic and toxicological sciences [7,8], drug discovery and development [9,10], and cosmetics [11], among others. cLC demonstrated great potential in academic research and industrial fields, being responsible for a considerable economic impact in the industry [12]. Several advantages of this technique may justify this trend, such as remarkable selectivity, robustness, speed, sensitivity, reproducibility, rigorous quantification, and the possibility of combination with diverse detectors and/or analytical instruments [13]. In addition, diverse types of chiral stationary phases (CSPs) have been developed over the years for analytical and preparative applications [14,15]. More than a hundred CSPs are currently commercially available [16]. The CSPs include polysaccharide derivatives, macrocyclic antibiotics, cyclodextrins (CD), proteins, crown-ethers, cyclofructans, synthetic polymers, molecularly-imprinted, Pirkle-type, and ion-exchange chiral selectors [17].

To follow the challenges in different research areas as well as the progress in instrumentation and technical advancement [18,19], the development of new CSPs continues to be a field of great interest among the scientific community [20,21,22].

In recent years, the computational study of chromatographic enantioseparation has become an important tool in understanding the chiral recognition mechanisms for diverse CSPs [23,24]. With the advances in computational studies, its successful application in the chromatographic field has progressively increased over time, allowing researchers to understand and anticipate experimental results. Computational studies can be very helpful in estimating the magnitude of the enantioselectivity, anticipating the elution order, predicting other classes of chiral analytes that can be separated, and establishing the more suitable chromatographic conditions, including the choice of the CSP, solvent system as mobile phase, chemical additives or modifiers, pH conditions, and column temperature. In addition, even when experimental results are available, computational studies can elucidate molecular mechanisms of enantioselectivity and provide solutions, proposing innovative procedures for specific challenges [25,26,27]. The elucidation of chiral recognition mechanisms is essential to clarify the enantioselective binding properties and the kind of noncovalent interactions between the chiral selector and enantiomers [28,29].

Chiral molecular recognition and enantioseparation involve the formation of transient diastereomeric complexes with different stability, through a sum of diverse types of interactions, including hydrogen-bond, ionic, π–π, ion–dipole, dipole–dipole, and Van der Waals interactions [30,31]. In addition to attractive interactions involved in forming enantiomer-selector complexes, steric repulsion, repulsive charges, and entry into chiral cavities within the selector also play key roles in some types of CSPs [32]. The presence of bulky moieties can prevent an enantiomer from accessing the chiral selector, giving rise to high enantioselectivity [33,34]. Many CSPs are known to incorporate bulky rigid elements to induce such high levels of enantioselectivity [35,36].

**Figure 1:**
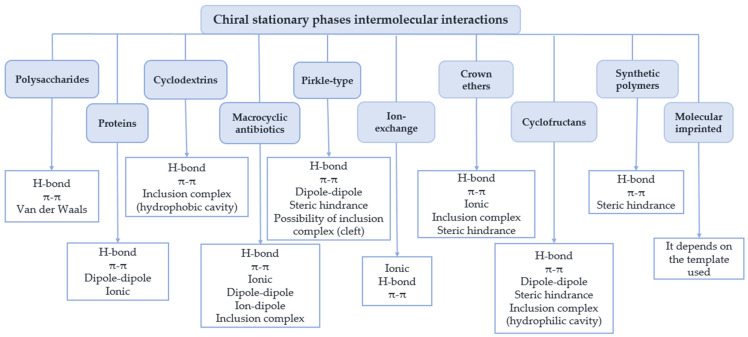
Intermolecular interactions associated with each type of chiral stationary phase (CSP).

Figure 1 summarizes the main intermolecular interactions between the chiral selector and the enantiomers to be separated for each type of CSP [32,37,38,39,40].

Nevertheless, despite numerous reported studies, the specific nature of the interaction between the selector for all types of CSPs and the enantiomers remains a challenge [28,41]. It is established that the forces acting on one enantiomer during the interaction with the selector may differ from those acting on the other enantiomer. Thus, the goal is to develop an approach that allows for the identification and quantification of the forces present [42]. Hence, a comprehensive understanding of chiral separation is pivotal because it allows the development of better chromatographic systems and elucidates fundamental concepts in chiral recognition [23]. The elucidation of chiral recognition mechanisms is also useful to guide structural modifications of the selectors to achieve a higher enantiomeric selectivity for a specific class or a wider range of enantiomers.

Chiral recognition is a type of molecular recognition involving the selective formation of transient complexes formed in a mixture of enantiomers [43]. The groundwork for understanding chiral recognition at a molecular level was laid by Bentley [44], who introduced rigid geometric models based on a biochemical and pharmacological perspective. From this concept, Easson and Stedman [45] developed a structural model to elucidate the differences in the biological activity of enantiomers. According to this model, enantioselectivity arises from the distinct interactions of a pair of enantiomers with biotargets, requiring at least three attractive contact points for chiral discrimination [28,45,46].

The “three-point model” was put aside for a period of time until Ogston [47] decided to reclaim it to explain the enzymatic decarboxylation of L-serine to glycine. Later, Topiol and Sabio [48] introduced the “four-contact point model”, which states that chiral recognition is associated with attractive or repulsive interactions between eight centers. Then, Mesecar and Koshland [49] introduced a “four-location model” where a minimum of four designed locations, such as four attachment sites or three attachment sites and a direction, is needed.

In the chromatography field, Dalgliesh [50] resorted to the “three-point model” to explain the chiral separation of aromatic amino acids using a CSP based on cellulose. This model of chiral recognition was later restated by Pirkle and coworkers [51,52,53], as follows: “Chiral recognition requires a minimum of three simultaneous interactions between the CSP and at least one of the enantiomers, with at least one of these interactions being stereochemically dependent.”

For some authors, the “three-point model” is considered simplistic, so it turns out not to be suitable for all selectors [54,55,56]. Actually, for macromolecules as chiral selectors, the mechanism can be much more complex [32,57]. Moreover, it can only be considered when selector-enantiomer interactions occur on only one side [58]. Additionally, it must always be taken into account that chiral recognition is a dynamic process and not a static process as it is often considered [59]. Nevertheless, this model has been widely used to design CSPs and for the rational study of the mechanisms associated with chiral discrimination [60,61,62].

A wide variety of tools has been described to shed light on the mechanisms involved in chiral molecular recognition and enantioseparation as well as elucidate the type of interactions that occur between a chiral selector and the enantiomers [63,64]. Nuclear magnetic resonance (NMR) spectroscopy, particularly nuclear Overhauser effect spectroscopy (NOESY) and rotating-frame Overhauser enhancement spectroscopy (ROESY), has proven to be a very valuable technique for determining the spatial proximity of functional groups of the selectors and enantiomers [65,66,67]. Additionally, ultraviolet (UV) spectroscopy, fluorimetry, Fourier transform, attenuated total reflectance infrared (IR) spectroscopy, and circular dichroism spectroscopy can be applied only for soluble selectors [64]. It is important to note that interactions will change with solvents, emphasizing the need for careful consideration when different solvents are used in certain studies and chromatographic separations [68,69]. X-ray crystallography provides the structures of the complexes between chiral selectors and enantiomers in a solid state; however, these differ from the structures in a solution [57,70,71]. Furthermore, immobilized chiral selectors adopt distinct conformations compared to their crystalline or solution states due to chemical modifications during immobilization and steric hindrance [72,73]. These constraints force structural adaptations to achieve optimal stability, directly impacting their chiral recognition properties [69,74].

The first example of molecular modeling regarding enantioseparation was the study developed by Armstrong et al. [75], where the formation of the inclusion complexes of β-CD was analyzed. Next, Weinstein et al. [76] introduced a molecular model to elucidate the intricacies of stereoselective interaction within a series of chiral secondary amines. Nevertheless, it was Lipkowitz et al. [77] who have notably led the computational studies in this field using quantum mechanics (QM), molecular mechanics (MM), molecular dynamics (MD), and Monte Carlo methods. Their extensive investigations have mainly focused on unravelling the enantioselectivity of various chiral selectors through the prediction/confirmation of the enantiomeric elution order [77] and, with a special focus, on understanding the chiral recognition mechanisms associated with chromatographic separations by CSPs developed by Pirkle’s group [78,79].

It is also important to highlight Scriba et al., who used MD and molecular modeling to examine binding thermodynamics and to visualize selector-analyte complex structures, contributing significantly to the understanding and development of computational studies in chiral molecular recognition [63,80]. This collective effort underscores the pivotal role of computational methods in advancing the understanding of chiral chromatography, offering invaluable insights into its underlying principles and applications.

In recent years, a number of fundamental reviews and book chapters on chiral recognition mechanisms in separation science have been published [23,25,32,57,63,64,69,80,81,82,83], focusing mainly on relevant examples across different types of CSPs. This review systematically compiles enantioseparation studies by cLC that incorporate both CSPs and computational analysis. The main aim of this review is to unfold the synergy between computational studies and cLC, to assess the relevance of the association of these two approaches, and to analyze current trends in the field. Special emphasis is placed on how computational advancements are reshaping the landscape of cLC, providing deeper insights and supporting the development of more efficient and selective separation strategies.

The key novelty of this review, in comparison with the existing literature, lies in the exhaustive compilation of studies integrating these two methodologies. This comprehensive survey offers a clear perspective on emerging trends over recent years, including year-by-year developments. It not only traces the evolution of computational applications but also examines various aspects of cLC, such as the types of CSPs employed, the composition of mobile phases, and the range of analytes investigated.

## 2. Molecular Modeling in Chiral Liquid Chromatography

In this review, a literature survey covering the reports on enantioseparation by cLC using CSPs and computational studies was conducted, covering recently published works (from 2015 to January 2025). The scientific compilation took place in January 2025 and was based on the PRISMA guidelines [84]. The identification of papers was conducted through a search on the SCOPUS database considering the following keywords or expressions: “chiral stationary phase AND computational OR docking OR molecular dynamics OR molecular modeling,” “enantioseparation AND computational OR molecular modeling AND liquid chromatography.”

**Figure 2:**
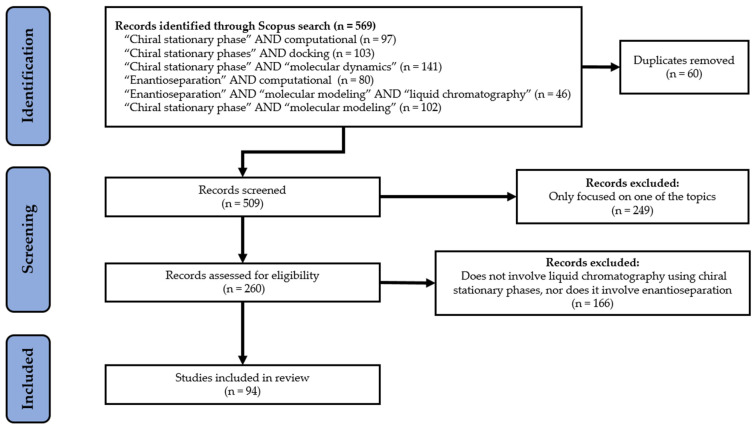
Flow diagram of literature search (n = number of scientific articles time frame: 2015–January 2025; database: SCOPUS).

Inclusion criteria for paper selection were works published as original articles, in English, that addressed the topics of this research. Studies describing other types of chromatography, not related to the separation of enantiomers, as well as studies not relevant to the topic, were excluded. All data collected were interpreted in a critical and impartial manner. The methodological path that led to the selection of the scientific articles included in this review was outlined according to the flowchart shown in Figure 2.

Through the comprehensive literature survey, 94 articles that included computational studies and enantioseparation by LC were found. Table 1 summarizes relevant information about the articles.

**Figure 3:**
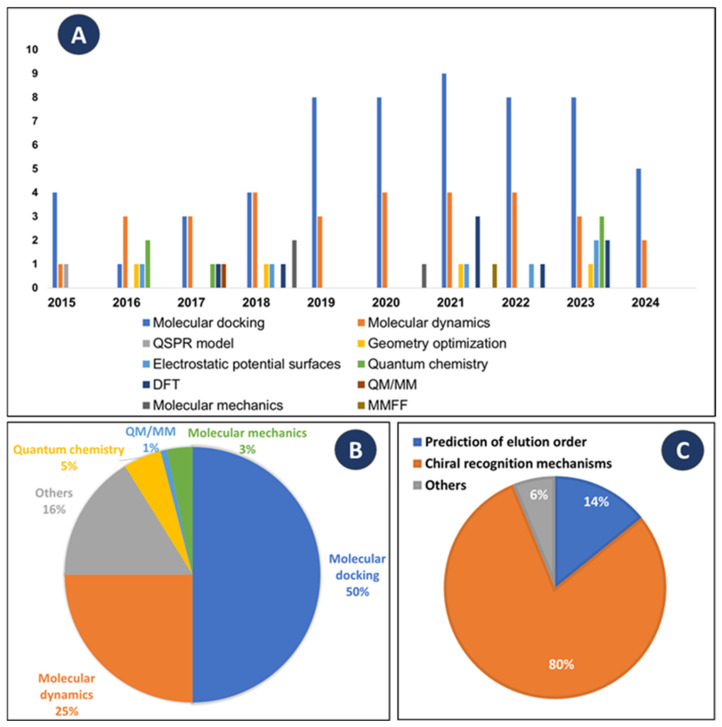
(**A**) Distribution of the selected computational studies between 2015 and 2024. (**B**) Distribution of the reported works considering the computational methods used. (**C**) Main aims of reported methods. DFT: Density functional theory; MMFF: Merck Molecular Force Field; QM/MM: Quantum mechanics/Molecular mechanics; QSPR: Quantitative structure-activity relationship; QM/MM: Quantum mechanics/Molecular mechanics.

A significant growth in research concerning both enantioseparation by cLC and computational studies is evident from 2015 to 2023, as illustrated in Figure 3A. Particularly, between 2019 and 2023, there is a notable spike in interest in this field, leading to an increase in article publications. Moreover, during 2024, 11 articles were published. These data emphasize the fact that the relationship between cLC and computational studies tends to grow over time. It was found that a diverse range of computational methods were employed.

As emphasized in Figure 3B, molecular docking emerges as the predominant computational method, accounting for 50% of the reported studies. The reasons that can justify this number are its user-friendly interface and light computational cost [177]. Following closely behind is MD (25% of the studies), also widely employed due to its ability to dynamically simulate the chiral separation process, including the solvent effect [178]. It is noteworthy that most of the studies include multiple computational approaches simultaneously to mitigate the limitations inherent to each technique.

Computational studies have been used to achieve various purposes, being extremely valuable in the enantioseparation field. As summarized in Figure 3C, the most common goal is the understanding of the chiral recognition mechanisms, accounting for 80% of the reported studies, followed by the prediction of the enantiomeric elution order, accounting for 14% of the studies.

**Figure 4:**
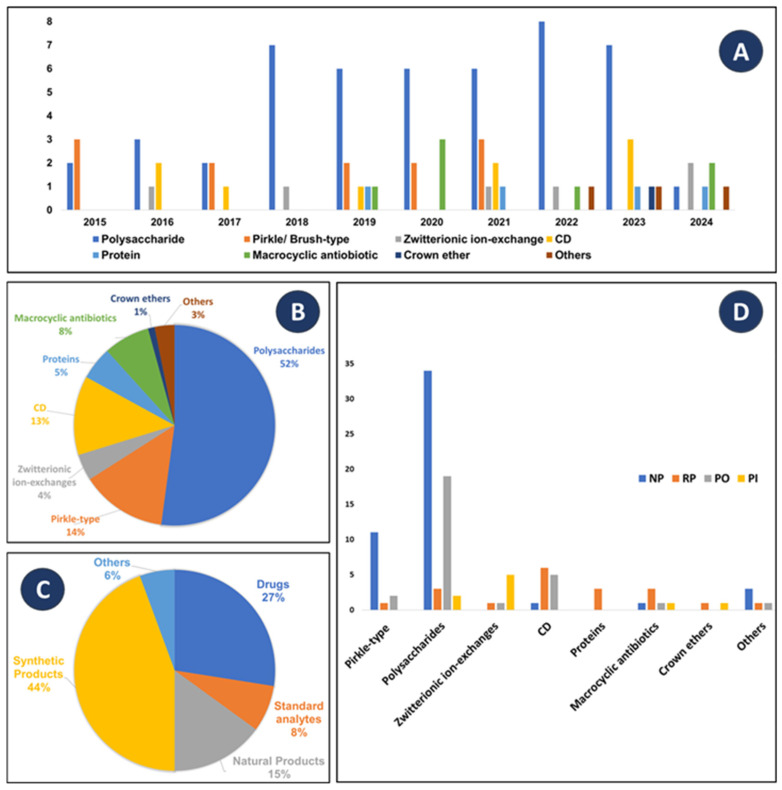
(**A**) Distribution of the reported studies considering the type of chiral stationary phases (CSPs) from 2015 to 2024; (**B**) Distribution regarding the types of CSPs. (**C**) Tested analytes in the studies reported in Table 1; (**D**) Modes of elution for each type of CSP. CD: Cyclodextrin; MP: Mobile phase; NP: Normal phase; PI: Polar ionic; PO: Polar organic; RP: Reversed-phase.

The success of an efficient enantioseparation is mostly determined by the chiral discriminative capability of the CSP [16]. Figure 4A summarizes the different types of CSPs reported from 2015 until the end of December 2024. In general, the most used CSPs, over the years, are polysaccharide-based and Pirkle-type. It is important to point out a notable increase in the number of studies describing both enantioseparation using polysaccharide-based CSPs and computational studies from 2018.

Polysaccharide-based CSPs are recognized as being the most successful and widely applied for both analytical and preparative enantioseparations [179]. The high recognition ability of polysaccharide derivatives, their abundance in nature, and their compatibility with various solvents are some of the reasons that can justify this trend [180]. The chiral recognition ability of polysaccharides is dependent on diverse structural features, including sugar units, stereogenic centers, type of linkage and its position, as well as the adjacent polymer chains [181]. The helical twist of the polymer backbone also has a key role in enantioselectivity [182]. Although polysaccharide-based selectors are complex polymeric structures and, consequently, computational studies are more demanding when compared with selectors based on small molecules, in the last years, there has been an increased interest in computational approaches for this type of CSPs. As shown in Figure 4B, CSPs based on polysaccharides are the most employed, accounting for 52% of the reported studies. The second most used and investigated CSPs are Pirkle-type (14% of the reported studies). Since the 1980s and for many years, Pirkle-type CSPs have been the most widely investigated concerning the knowledge of chiral recognition mechanisms [62]. The reasons are because they comprise small molecules as chiral selectors covalently bound to chromatographic support via a spacer, being apparently easier to study [15,183]. The distribution of the chiral molecules on the surface of the inert matrix allows easy access to the analytes, enabling numerous interactions between the chiral selector and the enantiomers [15].

The next analysis concerns the type of analytes tested in each of the studies (Figure 4C). A great variety of analytes can be enantioseparated using different CSPs, including synthetic products, drugs, natural products, standard analytes typically used to evaluate the performance of CSPs, among others. It is evident that certain compounds are more frequently tested for enantioseparation than others, especially synthetic products and drugs, accounting for 44% and 27% of the reported studies, respectively.

Another important aspect of the enantioseparation process is the mobile phase, which greatly influences the retention of the enantiomers, enantioselectivity, and resolution [184]. As shown in Figure 4D, different types of CSPs, including Pirkle-type, polysaccharide-based, and macrocyclic antibiotics, can be used in diverse elution modes (normal-phase (NP), reversed-phase (RP), polar organic (PO), polar-ionic (PI)), being compatible with a wide range of solvents as mobile phases. Although they can be used in different elution modes, it was found that Pirkle-type and polysaccharide-based CSPs were most used in NP and PO conditions. PI conditions were preferred for zwitterionic-ion exchange CSPs and, as expected, RP mode for protein-based, macrocyclic antibiotic-based, CD-based, and crown ether-based CSPs.

As examples, representative studies reported in Table 1 covering the computational approaches used in cLC will be explored in more detail to emphasize the benefit of the association of these two approaches. To select examples involving the most commonly used computational techniques, our primary criterion was to include studies encompassing different types of CSPs. This approach was intended to highlight the distinct molecular recognition mechanisms associated with each CSP type, which we consider a key factor in understanding the interplay between chromatographic performance and computational analysis.

### 2.1. Molecular Docking

Molecular docking is a computational technique used to predict the interaction geometry (pose) of two molecules, in this specific field, a chiral selector and enantiomers, based on their molecular structures [185]. It helps predict binding geometries and rank binding affinities of enantiomers with CSPs [23,25]. The main goal is to identify the most stable diastereomeric complex and assess enantiomer elution order based on interaction energy [23]. The docking process involves two stages: search phase and scoring phase [186]. The search phase predicts the conformation of enantiomers at the binding site [186,187]. This can be conducted with a rigid selector (lock and key model) or a flexible selector (induced-fit model) [188,189]. Grid points are used to map the selector’s binding sites, and an algorithm explores different enantiomer conformers to find the best binding site [23,25,190]. After obtaining several poses, the second phase is reached, the scoring phase, where poses are scored and ranked according to their interaction with the receptor using simple scoring functions [191]. The main types of scoring functions are force field-based, which calculate energies based on physics-based potentials, empirical ones, which use linear combinations of terms with adjustable coefficients optimized from experimental data, and knowledge-based, which use statistical descriptors based on crystallography data [192,193,194]. Although molecular docking has been the preferred computational approach to address the interaction between chiral selectors and enantiomeric pairs, these calculations are generally insufficient to provide thermodynamic and kinetic detail on the separation process since they rely on a static representation of the interaction between selector and enantiomeric pairs and often struggle to accurately describe the nature of the interaction between these, in particular when non-polar interactions are determinant for the interaction. Nevertheless, these simple calculations have consistently provided atomistic detail on selector:enantiomer interactions and helped explain experimental data.

**Figure 5:**
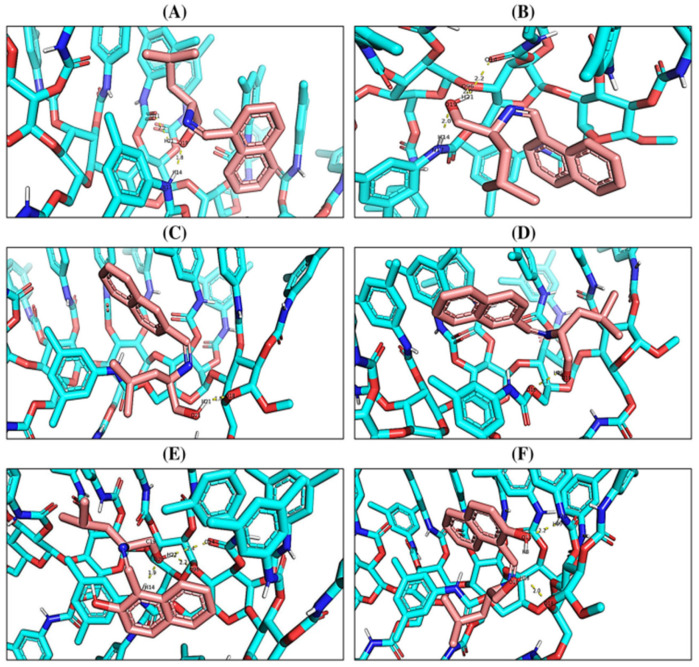
Docking poses of the enantiomers of 1-naphthaldimines (**A**,**B**), 2-naphthaldimines (**C**,**D**), and 2-hydroxynaphthaldimines (**E**,**F**) with tris(3,5-dimethylphenylcarbamate) cellulose-based CSP, respectively. The hydrogen-bond interactions are represented as yellow dotted lines, the analytes and the CSP are represented as light pink and cyan sticks, respectively. (Reprint with permission from [153], Copyright (2022) John Wiley and Sons).

Recently, Adhikari et al. [153] performed a molecular docking study of three naphthaldimine derivatives of leucinol on tris(3,5-dimethylphenylcarbamate) cellulose-based CSP Chiralcel OD-H^®^ to estimate the binding energies and conformations of the CSP-analyte complexes. The study included 100 docking runs, 25 × 105 energy evaluations, and 27.00 iterations using the Lamarckian genetic algorithm. Furthermore, the poses obtained were ranked using different scoring functions. The (*S*)-enantiomers of naphthaldimine derivatives exhibited stronger retention and binding affinity for the selector due to additional hydrogen-bonds, π–π and dipole–dipole interactions compared to the (*R*)-enantiomers. These differences in non-covalent interactions significantly enhanced enantioselectivity. The results obtained agreed with the experimental data of enantioseparation and elution order, where the (*R*)-enantiomer elutes before. In Figure 5, the docking poses of the enantiomers of each naphthaldimine derivative of leucinol are represented, alongside the main interactions responsible for chiral recognition (hydrogen-bond and π–π interactions) [153].

Another example of a molecular docking study was conducted by Dombi et al. [157], which focused on examining how apremilast (APR) enantiomers interact with human serum albumin (HSA) in a stereoselective manner. The docking data shed light on the interactions between APR enantiomers and HSA-based CSP, providing insights into the molecular mechanisms underlying their binding. The calculations were carried out using the Schrodinger suite, the system minimization was conducted by the OPLS3e force field, and the potential binding sites on HSA were identified using SiteMap. Flexible molecular docking was performed using the extra precision mode of Glide.

**Figure 6:**
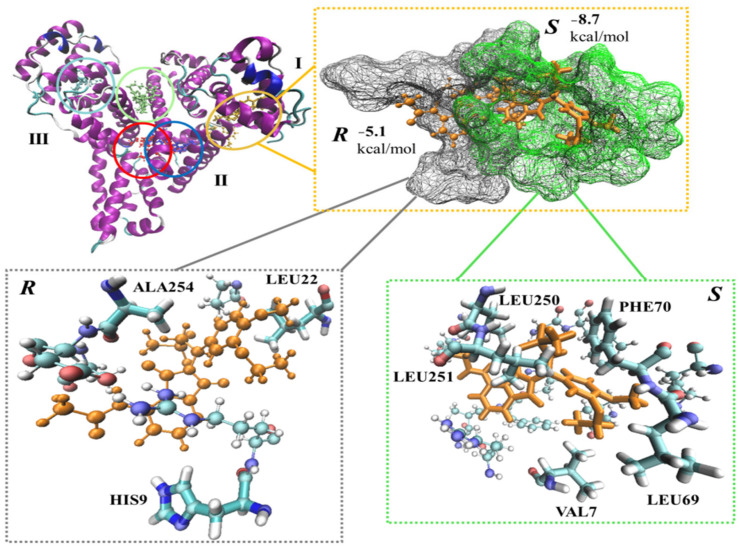
*S*-enantiomer and *R*-enantiomer of apremilast (APR) docked to binding sites of human serum albumin (HSA) (**top**, **left corner**). I, II, and III are HSA domains. The structures are highlighted in red, blue, lime, yellow, and cyan. The best binding pose for APR regarding the sites was chosen, along with the corresponding enantiomer (**top**, **right corner**). Amino acid residues within 2 Å of the ligands are highlighted (**bottom**) [157]. *R* and *S* enantiomers are colored orange.

The results showed that (*S*)-APR bound more strongly to the HSA, mainly due to an extra π-stacking interaction between this enantiomer and a Phe residue of HSA (Figure 6) [157].

**Figure 7:**
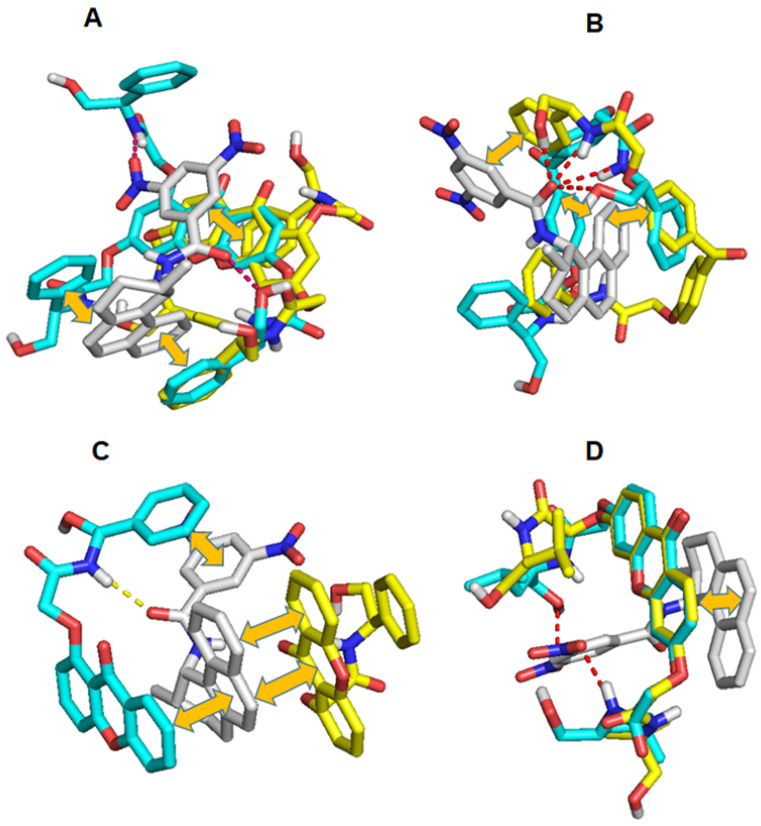
The most stable conformations of four analytes (**A**–**D**) docked onto (*S*,*S*)-Whelk-O1^®^ (grey). (*R*) and (*S*) enantiomers are represented as cyan and yellow sticks, respectively. Hydrogen-bond and π–π stacking interactions are represented as a red broken line and a yellow double arrow, respectively. (Reprint with permission from [195], Copyright (2021) John Wiley and Sons).

Phyo et al. [195] conducted a molecular docking study to understand the chromatographic results and to identify the chiral recognition mechanisms responsible for the enantioseparation of xanthones and benzophenones using (*S*,*S*)-Whelk-O1^®^ CSP. Additionally, the analysis of the interactions between the tested enantiomers and the chiral selector illustrated the role of the structural characteristics of the compounds for enantiodiscrimination. Docking data showed that the π–π stacking interactions established by the phenyl ring bonded to the stereogenic centers and the aromatic moiety of the selector were crucial for enantiorecognition. In Figure 7, it is possible to verify the interactions between the analytes (in this case, four of them as examples) and the selector responsible for enantioseparation [195].

### 2.2. Molecular Dynamics

MD simulations offer an effective way to study the dynamic interactions between enantiomers and CSPs in chromatographic separations, providing a more realistic representation than static calculations, such as in docking [39,59,141]. MD simulates molecular movement and interactions over time by solving Newton’s equations of motion for all atoms in a system, which often includes the CSP, solvent, and analytes [25,39,196]. The CSP is typically modeled in one of four ways: a dynamic amorphous silica plate, a fixed layer of silicon atoms, a polymer with limited mobility, and a loose selector molecule [86,197,198,199]. Solvent modeling can be conducted using explicit solvent (thousands of molecules), implicit solvent (dielectric solvent), or no solvent (vacuum) [96,129,200].

Both the analyte and selector can be treated as flexible or rigid molecules depending on the system’s requirements [201,202]. Those changes in the components of the system allow for conformational changes and exploration of different binding poses. MD simulations can reveal binding locations, intermolecular interactions responsible for diastereomeric complex formation, and the key forces driving enantioselectivity [141,178,203], and are thus powerful approaches to study dynamical and thermodynamic properties of complex systems. However, they are often time-consuming and computationally demanding, especially for large and heterogeneous systems, such as is the case for chromatographic ones. Aside from the complexity of the chemical composition of chromatographic systems, chromatographic separations generally take minutes to hours to occur, timescales that are unfeasible for atomistic simulations even with available computational power nowadays. Most common examples in the literature resort to the modeling of simple mixtures of the chiral separator and enantiomeric pairs in solution, but a few examples can be found where the modeling of materials functionalized with chiral separators and the mobile phase is attempted, allowing for a dynamic study of the interaction between chiral selectors and enantiomeric pairs [127]. Hence, despite the obvious challenges posed to the field, we can find several examples in literature where MD simulations provided valuable insights into the molecular basis of chiral separations [196,204].

**Figure 8:**
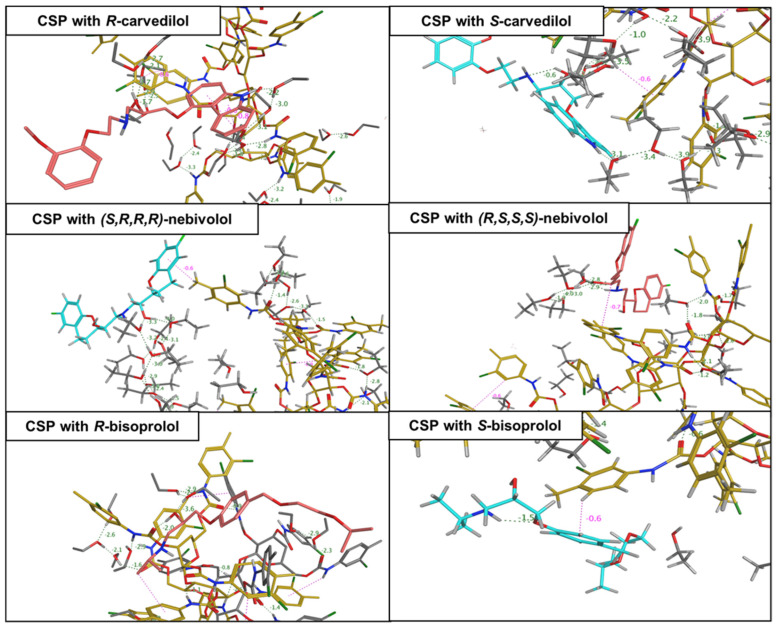
Binding interactions obtained from molecular dynamics (MD) simulations between cellulose-based chiral stationary phase (CSP) and three analytes, at 600 ps, adapted from [163]. Cellulose-based CSP is colored gray, *R* and *S* enantiomeric pairs are colored orange and cyan respectively.

For example, in a study developed by Saleh et al. [163], the enantioseparation, quantification, and chiral recognition mechanisms of five β-adrenergic blockers, namely bisoprolol, carvedilol, atenolol, metoprolol, and nebivolol, on a cellulose tris(3-chloro-4-methylphenyl carbamate column (Lux-Cellulose-2^®^), were investigated by molecular docking and MD. Docking studies identified the most stable complex for each enantiomer and the key interactions driving separation, while MD simulations were used to evaluate the stability of the enantiomer-CSP complex and confirmed the main interactions involved. Through a series of short MD simulations on simple systems solvated with ethanol, the authors could confirm the column’s separation capability for the analytes and their enantiomeric elution order: (*S*)-metoprolol > (*R*)-metoprolol; (*R*)-bisoprolol > (*S*)-bisoprolol; (*S*,*R*,*R*,*R*)-nebivolol > (*R*,*S*,*S*,*S*)-nebivolol; (*R*)-carvedilol > (*S*)-carvedilol; (*S*)-atenolol > (*R*)-atenolol, in line with experimental results. Furthermore, chiral recognition mechanisms were also identified, the main interactions being hydrogen-bond and π–π interactions. The (*R*)-atenolol showed stronger retention due to π–π stacking and two hydrogen-bonds, while the (*S*)-atenolol established one hydrogen-bond and one halogen bond. For carvedilol, the (*S*)-enantiomer was more retained due to two additional hydrogen bonds. For nebivolol, the (*S*,*R*,*R*,*R*)-enantiomer formed one hydrogen-bond and one π–π interaction, while the (*R*,*S*,*S*,*S*)-enantiomer established an extra hydrogen-bond, leading to stronger retention. Lastly, both bisoprolol enantiomers had π-alkyl interactions, but the (*S*)-enantiomer established two hydrogen bonds compared to one for the (*R*)-enantiomer. For example, in Figure 8 are represented the binding interactions of carvedilol, nebivolol, and bisoprolol with the CSP [163].

**Figure 9:**
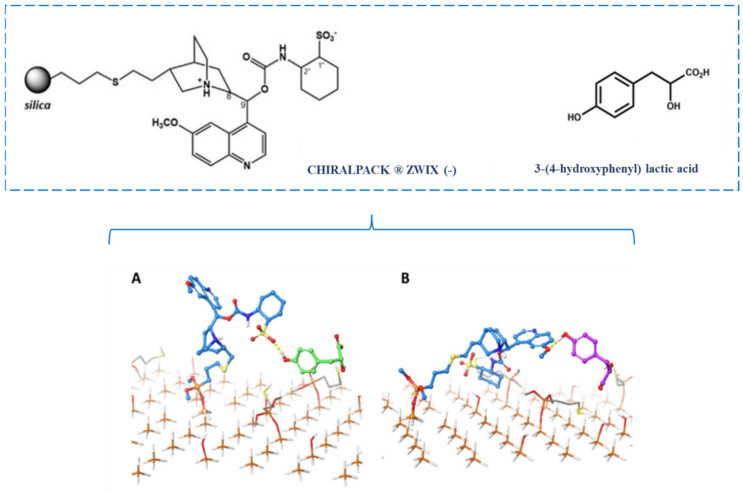
Examples of frames of hydrogen-bond interactions (yellow dashed lines) promoted by Chiralpack^®^ ZWIX (-) (cyan sticks) and the (*S*)-enantiomer (green sticks) (**A**) and (*R*)-enantiomer (magenta sticks) (**B**) of 3-(4-hydroxyphenyl) lactic acid. Adapted from [140]. (Reprint with permission from [140], Copyright (2021) Elsevier).

Varfaj et al. [140] used ab initio time-dependent density functional theory (DFT) simulations coupled with electronic circular dichroism to obtain the enantiomeric elution order under optimized mobile phase conditions. Additionally, MD simulations were carried out to determine the chiral recognition mechanisms associated with the enantioseparation of aromatic α-hydroxy acids with cinchona alkaloid-based zwitterionic CSP (ChiralPack^®^ ZWIX (-)) (Figure 9). The time-dependent DFT simulations were performed using the ωB7X-D3 density functional and the 6-311++G** basis set, and the 50 lowest energy electronic transitions of each optimized conformer were then used to calculate their theoretical electronic circular dichroism spectra. The MD simulations were performed in the canonical ensemble at 298 K, using the Desmond Molecular Dynamics System for 300 ns [140].

By benchmarking different theoretical electronic circular dichroism spectra obtained against an experimentally obtained one, the authors developed a computational strategy to determine the enantiomeric elution order: (*S*)-enantiomer first, followed by (*R*)-enantiomer. Subsequent MD simulations showed that the hydrogen bond interaction of the *p*-hydroxy group of 3-(4-hydroxyphenyl) lactic acid with the sulfonic acid moiety of the chiral selector (Figure 9) was essential for the retention and supported the experimental enantiomeric elution order [140].

**Figure 10:**
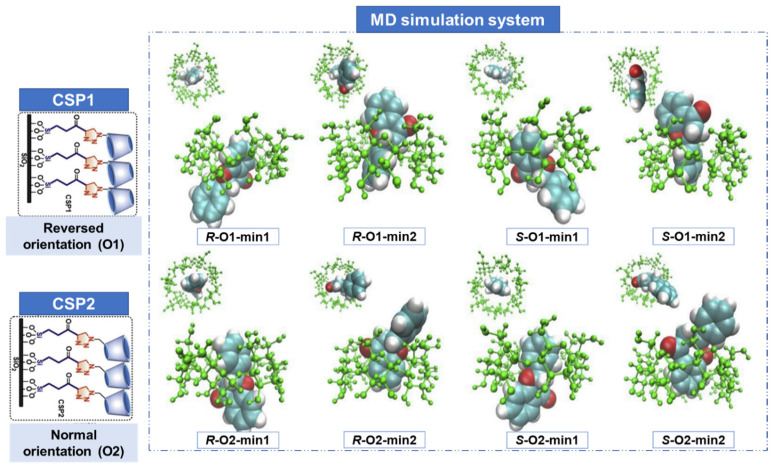
Snapshots from molecular dynamics (MD) simulation of the inclusion complexes of flavanone with β-CD-based chiral stationary phases (CSPs) with different orientations, reversed (CSP1) and normal (CSP2), adapted from [98]. (Reprint with permission from [98], Copyright (2017) Elsevier).

Another example was described by Wang et al. [98], which modeled the enantioseparation of a flavanone with β-CD-based CSPs with different orientations (normal and reversed) by MD simulations, using a 1:1 methanol/water mixture as mobile phase (Figure 10). The system was described with the CHARMM36 force field, β-CD were described by the CHARMM carbohydrate force field and parameters for the flavanones were derived from the CGenFF force field, and simulations were performed with the NAMD software. The simulations were carried out at 298 K and 1 atm conditions, controlled by Langevin Dynamics and Langevin Piston methods, respectively, and the dynamic simulation process was performed with a time step of 2.0 fs [98].

The results showed that the CD selector with the normal orientation (CSP2) allowed a better enantioseparation for almost 30 racemates, and the CD selector with the reversed orientation (CSP1) had a better resolution for analytes with polar functional groups in cyclic moieties. The MD simulations revealed inclusion complexes of the CSPs with different orientations (Figure 10); the main interactions responsible for enantioseparation, namely hydrophobic and hydrogen-bond interactions, were able to predict enantiomeric elution order and racemate resolution [98].

### 2.3. Other Computational Approaches

Although scarcer in the literature, QM calculations have also been reported to study chromatographic systems. QM is used to describe the properties of electrons and nuclei on a subatomic scale, allowing the study of molecular phenomena with electronic resolution [205,206]. The calculation of molecular properties with electronic resolution requires the resolution of the Schrodinger equation [206], in particular of the wavefunction of the system from which observable quantities of the system can be drawn [207]. DFT is the most common QM method for studying chromatographic systems [208]. The main disadvantage of these methods is that the systems under study cannot generally scale beyond a few hundred atoms, nor can their dynamic properties be simulated for periods longer than a few picoseconds. In particular, solvation must generally be addressed with simplistic implicit solvation models to reduce the number of atoms in the system and render QM calculations feasible. As such, they are generally employed to characterize interactions between selectors and enantiomeric pairs, complementing molecular docking studies. QM calculations have also been used to compute the spectroscopic properties, namely by using TD-DFT to calculate the circular dichroism spectra of enantiomeric pairs [140]. More recent developments in the calculation of UV circular dichroism spectra of biological molecules combining TD-DFT and range-separated density functionals, explicitly including solvent representation or accounting for the conformational molecular diversity, are also expanding the use of these techniques to more complex molecules [209,210,211]. These advancements should also enhance the application of QM methods for the characterization of enantiomeric mixtures, namely enantiomeric pairs of biological relevance.

For example, Núñez-Rico et al. [161] investigated the effectiveness of a homochiral metal-organic framework-based CSP, TAMOF-1, to separate a wide range of racemic mixtures of organic compounds. Using the semiempirical GFN2-xTB method and implicit solvation models, the study made use of the Conformer–Rotamer Ensemble Sampling Tool (CREST) program and enhanced sampling MD simulations in water to predict and rationalize the separation and capabilities of TAMOF-1. Additionally, DFT was employed to predict activation energy barriers for chiral inversion where low resolution occurred or where theoretical predictions differed from experimental results. The study accurately predicted the enantiomers elution order within the TAMOF-1′s channels, with computational predictions aligning with experimental results in over 90% of cases, highlighting TAMOF-1’s potential as an effective tool for chiral separations [161].

In a study developed by Protti et al. [162], time-dependent (TD)-DFT calculations were employed to investigate the absolute stereochemistry of the synthetic cathinones mephedone, methylone, and butylone and to infer about the enantiomeric elution order on a crown ether-based CSP. The molecules were submitted to geometry optimization and frequency calculations at the DFT level, using the B97-D3 functional, the def2-TZVP basis set, the density fitting approximation, and the IEFPCM solvation model for methanol. Then, TD-DFT calculations, using PBE0-13 functional combined with the def2-TZVPD basis set and IEFPCM solvation model for methanol, were performed to calculate UV and circular dichroism spectra, from which the results showed a (*R*) < (*S*)-enantiomeric elution order for mephedrone, methylone, and butylone [162].

Another example was described by Peluso et al. [103], which investigated the enantioseparation of atropisomeric fluorinated 3-arylthio-4,4’-bipyridines on cellulose-based CSPs, focusing on identifying additional interactions, particularly those involving electronic charge depletion regions as recognition sites at both chiral and achiral levels. The study aimed to assess the influence of pentafluorophenyl-centered π-hole on enantioseparation. Geometry optimization and calculation of electrostatic potential surfaces, as well as related parameters at the B3LYP/6-311G* level of theory, were performed. The computational data identified additional interactions, specifically stereoselective chalcogen and π-hole bonds. The evaluation of molecular properties also aided the design of analytes as probes and provided insights into the experimental chromatographic behaviors [103].

## 3. Conclusions

In chiral liquid chromatography (cLC), understanding enantiomeric discrimination mechanisms is of pivotal relevance. The key concept behind enantioseparation involves the formation of labile diastereomeric complexes, driven by various intermolecular interactions such as hydrogen-bond, ionic, π–π, ion–dipole, dipole–dipole, induced dipole–dipole, and Van der Waals interactions between the chiral selector and enantiomers. The Gibbs energy difference between these diastereomeric complexes is responsible for chiral recognition.

Computational methods, such as molecular docking and molecular dynamics (MD), offer valuable insights into these interactions, helping to investigate chiral recognition mechanisms, rationalize experimental enantiomeric elution orders, and optimize chromatographic and solvation systems. However, computational methods face challenges, including reliance on approximations that may not fully capture system complexities, high computational resource demands, and difficulties in accurately representing solvent effects. Other drawbacks include rigid or semi-flexible models, as they can overlook conformational changes during enantioseparation, and often computational data require validation, normally through experimental studies, as simplified in silico models may not fully align with the experimental outcomes.

Nevertheless, even with the associated disadvantages, integrating computational methods in cLC is essential as it allows a better understanding of the recognition mechanisms of chiral selectors and establishes a connection between theoretical insights and experimental data, thus serving as a complementary approach to LC experiments.

This systematic review, although grounded in a well-defined and robust methodological framework, as outlined in the flowchart presented in Figure 2, has certain limitations. It does not include a formal assessment of bias and may be subject to publication bias, which could influence the overall findings. Additionally, heterogeneity in study designs, sample types, and experimental conditions makes direct comparisons across studies difficult. Moreover, the rapid emergence of new cLC enantioseparation studies, including both CSPs and computational analysis, means that the conclusions drawn may quickly become outdated.

The data compiled in this review highlights the current trend of combining cLC enantioseparation and computational studies within the same work, due to the synergistic benefits of these two approaches.

## Figures and Tables

**Table 1:** Compilation of studies comprising both LC enantioseparation using chiral stationary phases (CSPs) and computational approaches.

Analytes	CSP	Elution Mode/Mobile Phase Solvents	Computa-tional Approach	Main Aims	Non-Covalent Interactions	Main Conclusions	Ref. Year
Dipeptides	Polysaccharide-based	-	Molecular docking	Study of elution order and chiral recognition mechanisms	HB, VDW, and electrostatic	Computational studies explained chromatographic results	[85] 2015
Alcohols	Pirkle-type	NP: Hex/ 2-PrOH	Molecular docking and MD	Study of elution order and chiral recognition mechanisms	HB and steric effect	HB and steric hindrance had a key role in enantioseparation	[86] 2015
Pidotimod	Polysaccharide-based	PI: MTBE/ ACN/TFA	Molecular docking	Study of chiral recognition mechanisms	HB and VDW	VDW interactions had a key role in enantioseparation	[87] 2015
Aromatic amines and α-hydroxy acids	Pirkle-type	NP: Hex/ 2-PrOH	Molecular docking	Study of chiral recognition mechanisms	HB, dipole–dipole, π–π stacking, and steric effect	Computational studies explained the interactions responsible for enantioseparation	[88] 2015
Chiral diarylme-thane	Pirkle-type	NP: 2-PrOH/Hex	QSPR model	Prediction of retention and separation factors	HB, ionic and steric effect	Computational studies predicted separation factors and elution order	[89] 2015
Drugs	Zwitterionic ion-exchange-type	PO: MeOH/THF	MD	Study of chiral recognition mechanisms	HB, π–π stacking, π-cation and ionic	CSPs acted as cation exchangers, and elution order was interpreted	[90] 2016
Pyrazoles	Polysaccharide-based	NP: Hex/EtOH or 2-PrOH PO: EtOH, 2-PrOH	MD	Study of solvents and temperature influence on separation	π–π stacking, HB, and hydrophobic	Computational studies predicted elution order and absolute configurations	[91] 2016
Polyhalo-genated 4,4′-bipyridines	Polysaccharide-based	NP: Hex/ 2-PrOH	Geometry optimiza-tion, computation of EPSs, and MD	Impact of the halogens on the chiral recognition mechanisms	Dipole–dipole, HB, π–π stacking, and XB	Electrostatic interactions had a key role in enantioseparation	[92] 2016
Drugs	CD-based	RP: H_2_O/ACN/ AcOH	QM	Investigation of chiral recognition mechanisms	Hydrophobic	Enantioseparation occurred due to different enantiomer binding affinities	[93] 2016
Drugs	CD-based	RP: H_2_O/MeOH or ACN	QM	Development, optimization, and validation of an LC-MS/MS method and study of chiral recognition mechanisms	Hydrophobic	The method was linear, accurate, and precise. *S*-POM established a more stable complex with the chiral selector	[94] 2016
Drugs	Polysaccharide-based	NP: Hex/ 2-PrOH or EtOH	Molecular docking	Study of elution order and chiral recognition mechanisms	HB	Elution order was *R*- prior to *S*-enantiomer	[95] 2016
Organic acids	Pirkle-type	NP: Hex/ 2-PrOH	Molecular docking, MD, and QM	Study of chiral recognition mechanisms	HB, π–π stacking, VDW, and steric effect	Interactions between the CSP and analytes were identified	[96] 2017
Chiral derivatives of xanthones	Pirkle-type	PO: MeOH/ACN	Molecular docking	Study of chiral recognition mechanisms	HB and π–π stacking	Good agreement between chromatographic and computational studies	[97] 2017
Dihydro-isoxazole, flavonoids, Troger’s base, amino acids, chromanols	CD-based	RP: H_2_O/MeOH or ACN	MD	Study of chiral recognition mechanisms	HB and Hydrophobic	Computational studies precisely predicted the elution order and resolution	[98] 2017
Butyro-lactones	Polysaccharide-based	NP: Hept/EtOH PO: MeOH	DFT and molecular docking	Enantiomers isolation and monitorization of elution order	HB	Computational studies confirmed elution order	[99] 2017
Flavonoids	Polysaccharide-based	PO: MeOH NP: Hept/ 2-PrOH	QM/MM and MD	Study of chiral recognition mechanisms	HB, π–π stacking and steric effect	Computational studies used as a pre-screening tool for choosing enantioseparation conditions	[100] 2017
Polyhalo-genated 4,4′-bipyri-dines, 2-nitro-1-aryletha-nols	Polysaccharide-based	NP: Hex/ 2-PrOH	MM and MD	Study of chiral recognition mechanisms	HB, π–π stacking, XB	Theoretical model allowed one to predict elution order	[101] 2018
Drugs	Polysaccharide-based	NP: Hex/EtOH or 2-PrOH or 1-PrOH	Molecular docking	Study of chiral recognition mechanisms	HB, hydrophobic π–π stacking	HB and hydrophobic interactions had a key role in enantioseparation	[102] 2018
Fluorinated 3-arylthio-4,4′-bi-pyridines	Polysaccharide-based	NP: Hex/ 2-PrOH, Hex/ 2-PrOH/MeOH PO: MeOH	MM, geometry Optimiza-tion, computation of EPSs, and MD	Study of chiral recognition mechanisms	Hydrophobic and π–π stacking	Computational studies designed analytes as probes and clarified chromatographic behaviors	[103] 2018
Polyhalo-genated 4,4′-bi-pyridines	Polysaccharide-based	NP: Hex/ 2-PrOH, Hex/ 2-PrOH/MeOH	MD	Study of chiral recognition mechanisms and elution order	π–π stacking, XB and hydrophobic	Computational studies showed higher sensitivity for weak XB detection and confirmed the elution order	[104] 2018
Coumarins	Polysaccharide-based	PO: MeOH/ACN	Molecular docking	Study of chiral recognition mechanisms	HB, π–π stacking	Computational studies confirmed the importance of HB and π–π interactions	[105] 2018
Drugs	Zwitterionic ion-exchange-type	PI: MeOH/THF/ DEA/FA	MD	Rationalization of enantiomeric elution order	-	The in silico model provided insights into enantiorecognition	[106] 2018
Drugs	Polysaccharide-based	NP: Hex/EtOH or 2-PrOH, Hex/FA/EtOH	TD-DFT and molecular docking	Study of chiral recognition mechanisms and elution order	HB, dipole–dipole, π–π stacking, and hydrophobic	Computational studies were in accordance with the experimental elution order	[107] 2018
Imizadoles	Polysaccharide-based	NP: Hex/EtOH or 2-PrOH or *n*-BuOH	Molecular docking	Study of chiral recognition mechanisms	HB, hydrophobic and π–π stacking	Computational studies predicted enantioseparation	[108] 2018
Alcohol esters	Polysaccharide-based	NP: Hex/MeOH PO: MeOH, EtOH, 2-PrOH	Molecular docking and MD	Investigation of solvent, ratio, and enantiomer structure on chiral recognition	HB, dipole–dipole, and π–π stacking	Computational studies predicted elution order and absolute configuration	[109] 2019
Chiral derivatives of xanthones	Protein-based	RP: ammonium acetate or sodium acetate or potassium phosphate buffers/ACN or MeOH or EtOH or 2-PrOH	Molecular docking	Study of chiral recognition mechanisms	HB and π–π stacking	Computational studies were in accordance with experimental results	[110] 2019
Triazoles	Polysaccharide-based	PO: ACN	Molecular docking	Study of chiral recognition mechanisms	XB, anion–π, HB, dipole–dipole, π–π stacking	Computational studies identified the chiral recognition mechanisms	[111] 2019
Drugs	Pirkle-type	NP: Hex/ 2-PrOH or 2-PrOH/AcOH	MD	Study of chiral recognition mechanisms	HB	Computational studies were in accordance with experimental elution order	[112] 2019
Drugs	Macrocyclic antibiotic-based	PI: MeOH/AcOH/TEA	Molecular docking	Identification of thermodynamic properties and study of chiral recognition mechanisms	HB, dipole–dipole, and electrostatic	Good agreement between computational studies and experimental results	[113] 2019
Indole alkaloids	Polysaccharide-based	NP: Hex/ 2-PrOH	Molecular docking	Study of chiral recognition mechanisms	Steric effect, π–π stacking, and HB	HB and π–π interactions were responsible for enantioseparation	[114] 2019
Chiral derivatives of xanthones, Troger’s base, alcohols, drugs	Pirkle-type	NP: Hex/EtOH or 2-PrOH, Hex/EtOH/ TEA RP:ACN/H_2_O/ TEA, PO: ACN, ACN/MeOH or EtOH	Molecular docking	Study of chiral recognition mechanisms	HB and π–π stacking	Computational studies identified the structural requirements for elucidation of chiral recognition	[115] 2019
Epoxide, aromatic ketones, flavonoids, drugs, biaryl compounds, amides, imidazolines	Polysaccharide-based	PO: MeOH, ACN NP: Hept/ 2-PrOH	MD	Development of a predictive method, considering both the dynamic nature of the process and the role of the solvent	HB, π–π stacking	Developed model was adequate for simulation of drug-CSP interactions	[59] 2019
Aromatic ketones, biaryl compounds, flavonoids, aromatic alcohols, drugs	CD and polysaccharide-based	RP: H_2_O/MeOH or ACN	Molecular docking	Study of chiral recognition mechanisms	Hydrophobic, HB, and π–π stacking	Computational studies showed a good agreement with experimental results	[116] 2019
Drugs	Polysaccharide-based	NP: Hex/ EtOH/ DEA	Molecular docking	Study of chiral recognition mechanisms	HB and π–π stacking	Computational studies explained chiral recognition mechanisms	[117] 2019
Troger’s base, epoxide, α-hydroxy ketones, alcohols, metal complexes, flavonoids	Polysaccharide-based	NP: Hex/ 2-PrOH	Molecular docking and MD	Study of chiral recognition mechanisms	HB and π-alkyl	Computational studies showed that polymer backbone conformation change was the main factor for enantioselectivity	[118] 2020
Drugs	Polysaccharide-based	NP: Hex/EtOH or 1-PrOH or 2-PrOH	Molecular docking	Study of chiral recognition mechanisms	HB, π–π stacking, hydrophobic	Computational studies agreed with experimental	[119] 2020
Indazole derivatives	Pirkle-type	NP: Hex	MD	Study of chiral recognition mechanisms	HB, π-cation and ionic, π–π stacking, and steric effect	In silico computational studies predicted enantioseparation	[120] 2020
Drugs	Polysaccharide-based	NP: Hex/EtOH or 2-PrOH/DEA	Molecular docking	Study of chiral recognition mechanisms	Hydrophobic, HB, π–π stacking, and steric effect	Computational studies agreed with experimental enantioselectivity	[121] 2020
Drugs	Polysaccharide-based	RP: Ammonium acetate buffer/MeOH	Molecular docking	Study of chiral recognition mechanisms	HB, π–π stacking, hydrophobic, and dipole–dipole	Computational studies were in accordance with chromatographic enantioselectivity	[122] 2020
Drugs	Macrocyclic antibiotics-based	RP: Ammonium acetate buffer/MeOH	Molecular docking	Study of chiral recognition mechanisms and elution order	π–π stacking	Computational studies showed that *R*-enantiomer binds stronger to the CSP, in accordance with experimental	[123] 2020
Dipeptide	Macrocyclic antibiotic-based	RP: MeOH/H_2_O	MD	Study of chiral recognition mechanisms	HB, π-cation, ionic, and π–π stacking	Computational studies allowed for the study of chiral recognition mechanism of teicoplanin-based CSP	[124] 2020
α-Hydroxy acid	Macrocyclic antibiotic-based	NP: Hept/ 2-PrOH/TFA	Molecular docking	Develop a vancomycin-based CSP and study of chiral recognition mechanisms	HB, π–π stacking	Computational studies identified the interactions between enantiomers and chiral selector	[125] 2020
α-Hydroxy ketones, aromatic alcohols, oxazolid-ones, alkyl-benzenes, polymers, organo-metallics	Pirkle-type	NP: Hex/ 2-PrOH	Molecular docking	Preparation of novel proline-based CSPs and study of chiral recognition mechanisms	HB, π–π stacking, dipole–dipole, and steric effect	HB and π–π interactions were critical for chiral discrimination	[126] 2020
Drugs, α-hydroxy ketones, flavonoids	Polysaccharide-based	NP: Hept/ 2-PrOH PO: MeOH	MD	Study of solid support role and prediction of enantiomeric elution order	Hydrophobic and HB	Computational studies predicted elution order, outperforming previous models	[127] 2020
Lysine derivatives	Polysaccharide-based	NP: Hex/ 2-PrOH or EtOH	Molecular docking	Study of chiral recognition mechanisms, influence of various alcohol modifiers and column temperature	HB, dipole–dipole and π–π stacking	Chiral separation process was enthalpy driven and chiral recognition mechanisms were identified	[128] 2020
Bipyridines	Polysaccharide-based	NP: Hex/ 2-PrOH	DFT, MD, and molecular docking	Study of chiral recognition mechanisms	HB, π–π stacking, and XB	Computational studies provided elution order in accordance with experimental data	[129] 2021
Drugs	Pirkle-type	NP: Hex/DCM/ MeOH	Molecular docking	Study of chiral recognition mechanisms	HB	Computational studies identified enantiorecognition mechanisms, *S*-enantiomer presented a better complex stability	[130] 2021
Drugs and α-hydroxy ketones	Protein-based	-	Molecular docking	Study of chiral recognition mechanisms	Hydrophobic, ionic, and HB	The chiral binding sites were located on cAGP	[131] 2021
Biaryl diol	Pirkle-type	NP: Hex/ 2-PrOH	MD	Study of chiral recognition mechanisms and elution process simulation	HB, π–π stacking	Computational studies showed that *S*-enantiomer was more retained	[132] 2021
Drugs	CD-based	RP: ACN/FA buffer	Molecular docking	Study of chiral recognition mechanisms	Hydrophobic and HB	HB interactions and inclusion complexation played a key role in chiral recognition	[133] 2021
Drugs	CD-based	NP: Hex/EtOH or 2-PrOH or 1-PrOH or 1-BuOH PO: MeOH, EtOH, ACN	Molecular docking	Development of a novel MDCPC CSP and study of chiral recognition mechanisms	HB, π–π stacking	HB, hydrophobic interactions, and inclusion complexation played a crucial role in enantioseparation	[134] 2021
Drugs	Polysaccharide-based	PO: ACN/MeOH	Molecular docking	Study of chiral recognition mechanisms	HB, π–π stacking, and hydrophobic	π–π, HB, hydrophobic interactions led to enantioselectivity	[135] 2021
Sulfoxides derivatives	Polysaccharide-based	PO: 2-PrOH NP: Hex/ 2-PrOH	MMFF, geometry optimiza-tion, conforma-tional and electrostatic potential analysis	Study of chiral recognition mechanisms	Dipole–dipole, π–π stacking, HB, and hydrophobic	Recognition model explained enantioselectivity and several aspects impacting enantioseparation	[136] 2021
Drugs	Polysaccharide-based	PO: MeOH, ACN	Molecular docking	Study of chiral recognition mechanisms	Electrostatic, HB, and π-sulfur	Inclusion complexes along with different interactions led to enantioselectivity	[137] 2021
Alcohols, biaryl diols, metal acetylacetonates, α-hydroxy ketones	Pirkle-type	NP: Hex/ 2-PrOH, Hex	Molecular docking	Study of chiral recognition mechanisms and rationalization of the inductive and steric effects of substituents on chiral discrimination	HB, π–π stacking and dipole–dipole	Computational studies provided a correlation between polarity, size and position of the substituent on the phenyl ring and chiral recognition	[138] 2021
Sulfoxides	Polysaccharide-based	PO: EtOH, 2-PrOH	TD-DFT and molecular docking	Study of enantiomeric elution order and chiral recognition mechanisms	HB and π–π stacking	Computational studies predicted elution order and established the importance of π–π stacking and enantiomer inclusion	[139] 2021
α-Hydroxy acids	Zwitterionic ion-exchange-type	PI: MeOH/AcOH or ACN/AcOH, ACN/AcOH or MeOH/AcOH or FA	TD-DFT and MD	Development of an LC-MS method and evaluate the enantiomeric elution order	HB	Computational studies predicted the elution order and unveiled the role of phenolic group in retention mechanism	[140] 2021
Epoxides, amines, flavonoids	Polysaccharide-based	NP: Hex/ 2-PrOH	MD	Elution order and separation factors prediction	HB, hydrophobic and π–π stacking	Computational studies predicted elution order and enantioselectivity (except for *trans*-stilbene)	[141] 2021
3-Arylthio-4,4′-bi-pyridines derivatives	Macrocyclic antibiotic-based	PO: MeOH, EtOH, MeOH/ACN	Molecular docking and MD	Study of chiral recognition mechanisms	Steric effect, HB, and π–π stacking	Computational studies provided insights into recognition mechanisms on TAG-based CSPs	[142] 2022
Drugs	Polysaccharide-based	NP:Hex/EtOH/AcOH or AcOH/DEA or AcOH/TEA	Molecular docking	Study of chiral recognition mechanisms	HB, hydrophobic and π–π stacking	Computational studies revealed the stereoselective interactions	[143] 2022
Drugs	Polysaccharide-based	RP: ACN/H_2_O/FA	Molecular docking	Study of chiral recognition mechanisms	HB, hydrophobic and π–π stacking	HB, hydrophobic interactions, and π–π stacking played a key role in chiral recognition	[144] 2022
4,4′-Bi-pyridines	Polysaccharide-based	NP: Hex/ 2-PrOH PO: MeOH	DFT and MD	Study of chiral recognition mechanisms	HB, π–π stacking, and hydrophobic	Computational studies identified the interactions responsible for enantioseparation	[145] 2022
Troger’s base, epoxides, α-hydroxy ketones, alcohols, flavonoids, sulfoxides, and drugs	Polysaccharide-based	NP: Hex/ 2-PrOH	Molecular docking	Study of chiral recognition mechanisms	HB, π–π stacking	Computational studies showed the contribution of different chitosan derivatives for enantioresolution	[146] 2022
Ferrocenes	Polysaccharide-based	NP: Hex/ 2-PrOH or 2-PrOH/MeOH PO: MeOH RP: MeOH/H_2_O	Molecular electron density isosurfaces and MD	Study of chiral recognition mechanisms	HB, hydrophobic	Analyte confinement in a hydrophobic cavity and HB interaction were essential for complex stabilization	[147] 2022
Esters	Polysaccharide-based	NP: MeOH/Hex	MD	Study of chiral recognition mechanisms	HB, π–π stacking and hydrophobic	HB interactions were the most important for enantioseparation	[148] 2022
Drugs and α-hydroxy acids	Zwitterionic ion-exchange-type	RP: MeOH/H_2_O/ TEA	Molecular docking	Study of chiral recognition mechanisms	HB, π–π stacking	Good agreement between computational and experimental	[149] 2022
Drugs	Polysaccharide-based	PO: ACN, MeOH	Molecular docking	Study of chiral recognition mechanisms	HB, hydrophobic and π–π stacking	Computational studies suggested that chiral recognition is due to different enantiomer binding poses	[150] 2022
Aromatic ketones, naphthols, indanol, cyclohexa-nols, esters, alcohols, sulfonami-des, oxazo-lidones, binaphthol	Synthetic polymer-based	NP: Hex/ 2-PrOH	Molecular docking	Study of chiral recognition mechanisms and correlation between stereocenters configuration/ position and chiral resolution	HB and π–π stacking	Computational studies showed that the strength and number of intermolecular HB played a key role in chiral discrimination	[151] 2022
Carboxami-de derivatives	Polysaccharide-based	-	Molecular docking	Computational studies of new carboxamide derivatives	-	All compounds presented good docking score	[152] 2022
Amines and alcohols	Polysaccharide-based	NP: PrOH/Hex	Molecular docking	Study of chiral recognition mechanisms	HB, π–π stacking, and dipole–dipole	Computational studies supported the experimental results	[153] 2023
Drugs	Polysaccharide-based	NP: Hex/EtOH/FA, Hex/ 2-PrOH/FA	Molecular docking	Study of chiral recognition mechanisms	HB, hydrophobic and steric effect	Computational methods showed that chiral recognition on CDCPC is an exothermic process driven by HB, hydrophobic interactions, and steric effects	[154] 2023
Ferrocenes	Polysaccharide-based	NP: Hex/ 2-PrOH	Electrostatic potential and MD	Study of chiral recognition mechanisms	HB, π–π stacking, dipole–dipole and XB	Computational studies showed that XBs participate in recognition mechanisms	[155] 2023
Drugs	CD-based	PO: MeOH, ACN	Molecular docking	Study of chiral recognition mechanisms	HB, hydrophobic and π–π stacking	Computational studies predicted elution order in several cases	[156] 2023
Drugs	Protein-based	RP: phosphate buffer and MeOH, EtOH, 1-PrOH, 2-PrOH, or ACN	Molecular docking	Study of chiral recognition mechanisms	π–π stacking	Computational studies detailed the characterization of the complex APR-HSA	[157] 2023
Isoxazolines, dansyl amino acids, flavonoids	CD-based	PO: MeOH, RP: MeOH/H_2_O, MeOH/TEAA	Molecular docking	Preparation of alkylinidazole CD-based CSPs, and study of chiral recognition mechanisms	HB, steric effects, and hydrophobic	Secondary hydroxyl groups, cavity size of CDs, hydrophobic and steric effects had a key role in chiral recognition	[158] 2023
Isoxazolines, dansyl amino acids, and flavonoids	CD-based	PO: MeOH, MeOH/TEAA RP: MeOH/H_2_O	Molecular docking	Preparation of CD-based CSPs and study of chiral recognition mechanisms	Inclusion complexation, HB, electrostatic, hydrophobic, and steric effects	Synergistic inclusion effect and rich electrostatic interaction sites were essential for chiral recognition	[159] 2023
Coumarins	Polysaccharide-based	PO: EtOH, 2-PrOH, BuOH	Molecular docking	Study of chiral recognition mechanisms	HB, π–π stacking, and hydrophobic	Computational studies predicted elution order and chiral recognition mechanisms	[160] 2023
Standard analytes	Metal–organic framework-based	PO:ACN NP: Hex/EtOH	QM, DFT, and geometry optimization	Prediction of the enantioseparation	-	Experimental data confirmed the models’ validity and the performance of TAMOF-1 columns	[161] 2023
Phenethyl-amines, tryptamines, cathinones	Crown ether-based	RP: MeOH/ H_2_O/AcOH PI: MeOH/ ACN, TEA, AcOH	QM and DFT	Development of enantioselective methods and investigation of absolute stereochemistry	-	Computational studies predicted elution order and chiral recognition mechanisms	[162] 2023
Drugs	Polysaccharide-based	NP: Hex/EtOH/ DEA	Molecular docking and MD	Study of chiral recognition mechanisms	HB and π–π stacking	Computational studies were in accordance with experimental	[163] 2023
Bipyridines derivatives	Polysaccharide-based	NP: Hex/2-PrOH, Hex/2-PrOH/MeOH	Electrostatic potential analysis and MD	Study of chiral recognition mechanisms	HB, π–π stacking, and VDW	HB, π–π, and VDW interactions had a key role in enantioselectivity	[164] 2023
Pidotimod	Polysaccharide-based	PI: ACN/FA/ MeOH/TFA, ACN/TFA/ 2-PrOH/FA	QM	Development and validation of an HPLC-MS method and study of chiral recognition mechanisms	HB	Validated method showed high sensitivity, and computational studies predicted enantioseparation	[165] 2023
Amino acid derivatives	Zwitterionic ion-exchange-type	RP: MeOH/AcOH/Ammonium acetate	MD	Study of chiral recognition mechanisms	HB, π–π stacking, and π-cation	Computational studies were in accordance with the experimental elution order and elucidate the chiral recognition mechanisms	[166] 2024
Dipeptides	Macrocyclic antibiotic-based	RP: MeOH/ Sodium acetate buffer	Molecular docking	Study of chiral recognition mechanisms	π–π stacking and hydrophobic	Computational studies not predicted elution order for all stereoisomers	[167] 2024
Amino acids	Macrocyclic antibiotic-based	RP: H_2_O/MeOH or EtOH or CH_3_CN	Molecular docking	Study of chiral recognition mechanisms	HB and π–π stacking	Computational studies elucidated selector and analyte interactions	[168] 2024
Drugs	Polysaccharide-based	NP: Hex/EtOH/ DEA	Molecular docking	Study of chiral recognition mechanisms	HB and π–π stacking	Computational studies allowed understanding the chiral recognition mechanisms	[169] 2024
Drugs	Pillar [5] arene-based mesoporous silica	NP: Hex/ 2-PrOH RP: MeOH or ACN/H_2_O	Molecular docking	Preparation of new CSPs and study of chiral recognition mechanisms	HB and π–π stacking	Computational studies allowed understanding the chiral recognition mechanisms	[170] 2024
Allantoin	Zwitterionic ion-exchange-type	RP: ACN/MeOH/ H_2_O/AcOH	MD	Study of the chiral recognition mechanisms	HB and π–π stacking	Computational studies demonstrated HB was the key interaction for enantioselectivity	[171] 2024
Drugs and synthetic products	Protein-based	RP: Potassium phosphate buffer/ACN or 2-PrOH	Molecular docking	Study of the chiral recognition mechanisms	HB and π–π stacking	Computational studies suggested that drugs competition occurred in both HSA sites I and II	[172] 2024
1,4-Dihydro-pyrimidine derivatives	Polysaccharide-based	NP: Hex/ 2-PrOH	Molecular docking	Determination of kinetic properties and recognition mechanisms	π–π, π-alkyl, and π-halogen	Computational studies predicted enantiomers elution order	[173] 2024
Quinoline alkaloid isomers	CD-based	RP: MeOH/ACN/ 2-PrOH-H_2_O or ACN-ammonium formate buffer	Molecular docking	Synthesis of novel CSPs and study of chiral recognition mechanisms	HB and hydrophobic	Computational studies confirmed the advantages of the CSP supramolecular structure	[174] 2024
Drugs	Protein-based	RP: Phosphate buffer/MeOH or EtOH or 2-PrOH	Molecular docking	Study of the chiral recognition mechanisms	HB, π–π, and alkyl-π	Computational studies allowed understanding the chiral recognition mechanisms	[175] 2024
Standard compounds	Pirkle-type	NP: Hex/CHCl_3_	Molecular docking	Study of the chiral recognition mechanisms	HB and π–π stacking	Computational studies revealed the relevance chiral selector bent structure and cleft-like cavity for chiral recognition	[176] 2024

ACN: Acetonitrile; AcOH: Acetic acid; APR: Apremilast; BuOH: Butanol; cAGP: Chicken α1-acid glycoprotein; CD: Cyclodextrin; CDCPC: Cellulose tris (3,5-dichlorophenylcarbamate; CSP: Chiral stationary phase; DEA: Diethylamine; DCM: Dichloromethane; DFT: Density functional theory; EPS: Electrostatic potentials surfaces; EtOH: Ethanol; FA: Formic acid; HB: Hydrogen bonds; Hept: Heptane; Hex: Hexane; HPLC: High performance liquid chromatography; HSA: Human serum albumin; LC: Liquid chromatography; MD: Molecular dynamics; MDCPC: 3,5-Dichloro-phenylcarbamated mono-6-ethylenediamine-β-cyclodextrin; MeOH: Methanol; MMFF: Merck molecular force field; MS: Mass spectrometry; MTBE: Methyl-*tert*-butyl-ether; NP: Normal phase; PI: Polar ionic; PO: Polar organic; POM: Pomalidomide; PrOH: Propanol; QM: Quantum mechanics; QM/MM: Quantum mechanics/molecular mechanics; QSPR: Quantitative structure property relationship; RP: Reversed-phase; TAG: Teicoplanin aglycone; TD-DFT: Time-dependent density functional theory; TEA: Triethylamine; TEAA: Triethyl ammonium acetate; TFA: Trifluoroacetic acid; THF: Tetrahydrofuran; UHPLC-UV: Ultra-high performance liquid chromatography; VDW: Van der Waals; XB: Halogen bond.

## Data Availability

No new data were created or analyzed in this study. Data sharing is not applicable to this article.

## References

[1] Rocco A., Aturki Z., Fanali S. (2013). Chiral separations in food analysis. TrAC Trends Anal. Chem..

[2] Alvarez-Rivera G., Bueno M., Ballesteros-Vivas D., Cifuentes A. (2020). Chiral analysis in food science. TrAC Trends Anal. Chem..

[3] Sardella R., Ianni F., Marinozzi M., Macchiarulo A., Natalini B. (2017). Laboratory-scale preparative enantioseparations of pharmaceutically relevant compounds on commercially available chiral stationary phases for HPLC. Curr. Med. Chem..

[4] Nie Y., Liu X., Yang X., Zhao Z. (2013). Recent application of chiral liquid chromatography–tandem mass spectrometric methods for enantiomeric pharmaceutical and biomedical determinations. J. Chromatogr. Sci..

[5] Ribeiro A.R., Maia A.S., Ribeiro C., Tiritan M.E. (2020). Analysis of chiral drugs in environmental matrices: Current knowledge and trends in environmental, biodegradation and forensic fields. TrAC Trends Anal. Chem..

[6] Barreiro J.C., Tiritan M.E., Cass Q.B. (2021). Challenges and innovations in chiral drugs in an environmental and bioanalysis perspective. TrAC Trends Anal. Chem..

[7] Coelho M.M., Fernandes C., Remião F., Tiritan M.E. (2021). Enantioselectivity in drug pharmacokinetics and toxicity: Pharmacological relevance and analytical methods. Molecules.

[8] Almeida A.S., Silva B., Pinho P.G., Remião F., Fernandes C. (2022). Synthetic cathinones: Recent developments, enantioselectivity studies and enantioseparation methods. Molecules.

[9] Miller L., Potter M. (2008). Preparative chromatographic resolution of racemates using HPLC and SFC in a pharmaceutical discovery environment. J. Chromatogr. B.

[10] Leek H., Andersson S. (2017). Preparative scale resolution of enantiomers enables accelerated drug discovery and development. Molecules.

[11] Li M., Luo S., Di X., Cui Y. (2022). Ultrasound-assisted extraction coupling to high performance liquid chromatography for enantiomerically quantitative analysis of two preservatives in cosmetics and the potentially cytotoxic study. Microchem. J..

[12] Blehaut J., Franco P., Zhang T., Lang E., Valery E., Marcoux J.F. (2012). 9.17 Industrial applications of chiral chromatography. Compr. Chirality.

[13] Snyder L.R., Kirkland J.J., Dolan J.W. (2011). Introduction to Modern Liquid Chromatography.

[14] Pinto M.M., Fernandes C., Tiritan M.E. (2020). Chiral separations in preparative scale: A medicinal chemistry point of view. Molecules.

[15] Tang M., Zhang J., Zhuang S., Liu W. (2012). Development of chiral stationary phases for high-performance liquid chromatographic separation. TrAC Trends Anal. Chem..

[16] Zhang J.H., Xie S.M., Yuan L.M. (2022). Recent progress in the development of chiral stationary phases for high-performance liquid chromatography. J. Sep. Sci..

[17] Teixeira J., Tiritan M.E., Pinto M.M., Fernandes C. (2019). Chiral stationary phases for liquid chromatography: Recent developments. Molecules.

[18] Nazario C.E., Silva M.R., Franco M.S., Lancas F.M. (2015). Evolution in miniaturized column liquid chromatography instrumentation and applications: An overview. J. Chromatogr. A.

[19] Fekete S., Kohler I., Rudaz S., Guillarme D. (2014). Importance of instrumentation for fast liquid chromatography in pharmaceutical analysis. J. Pharm. Biomed. Anal..

[20] Liu H., Wu Z., Chen J., Wang J., Qiu H. (2023). Recent advances in chiral liquid chromatography stationary phases for pharmaceutical analysis. J. Chromatogr. A.

[21] Fernandes C., Lima R., Pinto M.M., Tiritan M.E. (2022). Chromatographic supports for enantioselective liquid chromatography: Evolution and innovative trends. J. Chromatogr. A.

[22] Fernandes C., Teixeira J., Pinto M.M., Tiritan M.E. (2021). Strategies for preparation of chiral stationary phases: Progress on coating and immobilization methods. Molecules.

[23] Sardella R., Camaioni E., Macchiarulo A., Gioiello A., Marinozzi M., Carotti A. (2020). Computational studies in enantioselective liquid chromatography: Forty years of evolution in docking-and molecular dynamics-based simulations. TrAC Trends Anal. Chem..

[24] Peluso P., Chankvetadze B. (2024). Recent developments in molecular modeling tools and applications related to pharmaceutical and biomedical research. J. Pharm. Biomed. Anal..

[25] Peluso P., Dessì A., Dallocchio R., Mamane V., Cossu S. (2019). Recent studies of docking and molecular dynamics simulation for liquid-phase enantioseparations. Electrophoresis.

[26] Sardella R., Ianni F., Macchiarulo A., Pucciarini L., Carotti A., Natalini B. (2018). Elucidation of the chromatographic enantiomer elution order through computational studies. Mini Rev. Med. Chem..

[27] Dallocchio R., Dessì A., Sechi B., Peluso P. (2023). Molecular dynamics simulations of amylose-and cellulose-based selectors and related enantioseparations in liquid phase chromatography. Molecules.

[28] Peluso P., Chankvetadze B. (2022). Recognition in the domain of molecular chirality: From noncovalent interactions to separation of enantiomers. Chem. Rev..

[29] Mcconnell O., Bach A., Balibar C., Byrne N., Cai Y., Carter G., Chlenov M., Di L., Fan K., Goljer I. (2007). Enantiomeric separation and determination of absolute stereochemistry of asymmetric molecules in drug discovery—Building chiral technology toolboxes. Chirality.

[30] Jin M.Y., Zhen Q., Xiao D., Tao G., Xing X., Yu P., Xu C. (2022). Engineered non-covalent π interactions as key elements for chiral recognition. Nat. Commun..

[31] Chen W., Fu L., Zhu Z., Liu J., Cheng L., Xu Z., Dong H., Ma J., Li Y., Fan X. (2023). Synergistic regulation of intermolecular interactions to control chiral structures for chiral recognition. Chin. Chem. Lett..

[32] Lämmerhofer M. (2010). Chiral recognition by enantioselective liquid chromatography: Mechanisms and modern chiral stationary phases. J. Chromatogr. A.

[33] Cavazzini A., Nadalini G., Dondi F., Gasparrini F., Ciogli A., Villani C. (2004). Study of mechanisms of chiral discrimination of amino acids and their derivatives on a teicoplanin-based chiral stationary phase. J. Chromatogr. A.

[34] He S., He Y., Cheng L., Wu Y., Ke Y. (2018). Novel chiral ionic liquids stationary phases for the enantiomer separation of chiral acid by high-performance liquid chromatography. Chirality.

[35] Siret L., Tambuté A., Bégos A., Rouden J., Caude M. (1991). Steric hindrance influence on the enantiorecognition ability of tyrosine-derived chiral stationary phases. Chirality.

[36] Fernandes C., Phyo Y.Z., Silva A.S., Tiritan M.E., Kijjoa A., Pinto M.M. (2018). Chiral stationary phases based on small molecules: An update of the last 17 years. Sep. Purif. Rev..

[37] Aboul-Enein H.Y., Kannappan V., Kanthiah S. (2022). Impact of cyclofructan derivatives as efficient chiral selector in chiral analysis: An overview. Chirality.

[38] Maier N.M., Lindner W. (2007). Chiral recognition applications of molecularly imprinted polymers: A critical review. Anal. Bioanal. Chem..

[39] De Gauquier P., Vanommeslaeghe K., Vander Heyden Y., Mangelings D. (2022). Modelling approaches for chiral chromatography on polysaccharide-based and macrocyclic antibiotic chiral selectors: A review. Anal. Chim. Acta.

[40] Junior F.M., Junior J.M. (2023). Absolute configuration from chiroptical spectroscopy. Chiral Separations and Stereochemical Elucidation: Fundamentals, Methods, and Applications.

[41] Schneider H.J. (1991). Mechanisms of molecular recognition: Investigations of organic host–guest complexes. Angew. Chem. Int. Ed..

[42] Lipkowitz K.B. (1995). Theoretical studies of type II–V chiral stationary phases. J. Chromatogr. A.

[43] Pirkle W.H., Pochapsky T.C. (1989). Considerations of chiral recognition relevant to the liquid chromatography separation of enantiomers. Chem. Rev..

[44] Bentley R. (2003). Diastereoisomerism, contact points, and chiral selectivity: A four-site saga. Arch. Bichem Biophys..

[45] Easson L.H., Stedman E. (1933). Studies on the relationship between chemical constitution and physiological action: Molecular dissymmetry and physiological activity. Biochem. J..

[46] Mikhael S., Abrol R. (2019). Chiral graphs: Reduced representations of ligand scaffolds for stereoselective biomolecular recognition, drug design, and enhanced exploration of chemical structure space. Chem. Med. Chem..

[47] Ogston A.G. (1948). Interpretation of experiments on metabolic processes, using isotopic tracer elements. Nature.

[48] Topiol S., Sabio M. (1989). Interactions between eight centers are required for chiral recognition. J. Am. Chem. Soc..

[49] Mesecar A.D., Koshland Jr D.E. (2000). A new model for protein stereospecificity. Nature.

[50] Dalgliesh C.E. (1952). 756. The optical resolution of aromatic amino-acids on paper chromatograms. J. Chem. Soc..

[51] Pirkle W.H., Pochapsky T.C. (1986). A new, easily accessible reciprocal chiral stationary phase for the chromatographic separation of enantiomers. J. Am. Chem. Soc..

[52] Pirkle W.H., Pochapsky T.C. (1987). Chiral molecular recognition in small bimolecular systems: A spectroscopic investigation into the nature of diastereomeric complexes. J. Am. Chem. Soc..

[53] Pirkle W.H., Finn J., Morrison J.D. (1983). 6-Separation of Enantiomers by Liquid Chromatographic Methods. Asymmetric Synthesis.

[54] Davankov V.A. (1997). The nature of chiral recognition: Is it a three-point interaction?. Chirality.

[55] Booth T.D., Wahnon D., Wainer I.W. (1997). Is chiral recognition a three-point process?. Chirality.

[56] Kafri R., Lancet D. (2004). Probability rule for chiral recognition. Chirality.

[57] Scriba G.K. (2012). Chiral recognition mechanisms in analytical separation sciences. Chromatographia.

[58] Jozwiak K., Moaddel R., Ravichandran S., Plazinska A., Kozak J., Patel S., Yamaguchi R., Wainer I.W. (2008). Exploring enantiospecific ligand–protein interactions using cellular membrane affinity chromatography: Chiral recognition as a dynamic process. J. Chromatogr. B.

[59] Wang X., House D.W., Oroskar P.A., Oroskar A., Oroskar A., Jameson C.J., Murad S. (2019). Molecular dynamics simulations of the chiral recognition mechanism for a polysaccharide chiral stationary phase in enantiomeric chromatographic separations. Mol. Phys..

[60] Davankov V.A. (2022). 50 years of chiral liquid Chromatography. How it started. J. Chromatogr. A.

[61] Tiritan M.E., Pinto M.M., Fernandes C. (2023). Pirkle type. Chiral Separations and Stereochemical Elucidation: Fundamentals, Methods, and Applications.

[62] Fernandes C., Tiritan M.E., Pinto M.M. (2013). Small molecules as chromatographic tools for HPLC enantiomeric resolution: Pirkle-type chiral stationary phases evolution. Chromatographia.

[63] Scriba G.K. (2019). Chiral recognition in separation sciences. Part II: Macrocyclic glycopeptide, donor-acceptor, ion-exchange, ligand-exchange and micellar selectors. TrAC Trends Anal. Chem..

[64] Scriba G.K. (2024). Update on chiral recognition mechanisms in separation science. J. Sep. Sci..

[65] Li G.W., Wang X.J., Cui D.D., Zhang Y.F., Xu R.Y., Shi S.H., Liu L.T., Wang M.C., Liu H.M., Lei X.X. (2020). Azaheterocyclic diphenylmethanol chiral solvating agents for the NMR chiral discrimination of alpha-substituted carboxylic acids. RSC Adv..

[66] Marta T.B., Argondizzo A.C., da Silva Oliboni R., Silva M.S. (2023). NMR chiral recognition of lipoic acid by cinchonidine CSA: A stereocenter beyond the organic function. Chirality.

[67] Silva M.S. (2017). Recent advances in multinuclear NMR spectroscopy for chiral recognition of organic compounds. Molecules.

[68] Uccello-Barretta G., Vanni L., Balzano F. (2010). Nuclear magnetic resonance approaches to the rationalization of chromatographic enantiorecognition processes. J. Chromatogr. A.

[69] Scriba G.K. (2016). Chiral recognition in separation science–an update. J. Chromatogr. A.

[70] Pirkle W.H., Murray P.G., Wilson S.R. (1996). X-ray crystallographic evidence in support of a proposed chiral recognition mechanism. J. Org. Chem..

[71] Chankvetadze B., Burjanadze N., Pintore G., Bergenthal D., Bergander K., Mühlenbrock C., Breitkreuz J., Blaschke G. (2000). Separation of brompheniramine enantiomers by capillary electrophoresis and study of chiral recognition mechanisms of cyclodextrins using NMR-spectroscopy, UV spectrometry, electrospray ionization mass spectrometry and X-ray crystallography. J. Chromatogr. A.

[72] Okamoto Y., Ikai T. (2008). Chiral HPLC for efficient resolution of enantiomers. Chem. Soc. Rev..

[73] Ward T.J., Ward K.D. (2012). Chiral separations: A review of current topics and trends. Anal. Chem..

[74] Haginaka J. (2001). Protein-based chiral stationary phases for high-performance liquid chromatography enantioseparations. J. Chromatogr. A.

[75] Armstrong D.W., Ward T.J., Armstrong R.D., Beesley T.E. (1986). Separation of drug stereoisomers by the formation of β-cyclodextrin inclusion complexes. Science.

[76] Weinstein S., Leiserowitz L., Gil-Av E. (1980). Chiral secondary amides. 2. Molecular packing and chiral recognition. J. Am. Chem. Soc..

[77] Lipkowitz K.B. (2001). Atomistic modeling of enantioselection in chromatography. J. Chromatogr. A.

[78] Lipkowitz K.B. (1994). Theoretical studies of brush-type chiral stationary phases. J. Chromatogr. A.

[79] Lipkowitz K.B., Baker B. (1990). Computational analysis of chiral recognition in Pirkle phases. Anal. Chem..

[80] Scriba G.K. (2019). Chiral recognition in separation sciences. Part I: Polysaccharide and cyclodextrin selectors. TrAC Trends Anal. Chem..

[81] Meng M., Wang L., Reuschel S. (2015). Computational approaches in developing accelerated chiral liquid chromatography techniques for mass spectral assays. Advanced LC-MS Applications in Bioanalysis.

[82] Scriba G.K. (2019). Recognition mechanisms of chiral selectors: An overview. Chiral Sep. Meth Prot..

[83] Ikai T., Okamoto Y. (2010). Preparation and chiral recognition of polysaccharide-based selectors. Chiral Recognition in Separation Methods: Mechanisms and Applications.

[84] Page M.J., Mckenzie J.E., Bossuyt P.M., Boutron I., Hoffman T.C., Mulrow C.D., Shamseer L., Tetzlaff J.M., Akl E.A., Brennan S.E. (2021). The PRISMA 2020 statement: An updated guideline for reporting systematic reviews. BMJ.

[85] Ali I., Haque A., Al-Othman Z.A., Al-Warthan A., Asnin L. (2015). Stereoselective interactions of chiral dipeptides on amylose based chiral stationary phases. Sci. China Chem..

[86] Ashtari M., Cann N.M. (2015). The docking of chiral analytes on proline-based chiral stationary phases: A molecular dynamics study of selectivity. J. Chromatogr. A.

[87] Dou X., Su X., Wang Y., Chen Y., Shen W. (2015). Studies on pidotimod enantiomers with Chiralpak-IA: Crystal structure, thermodynamic parameters and molecular docking. Chirality.

[88] He Z.J., Song H., Zhang Y.W., Wang D.C., Yao S. (2015). Chiral stationary phases and their relationship with enantiomer structures in enantioseparation research of analytical laboratory. J. Mex. Chem. Soc..

[89] Hu G.X., Luo C.C., Pan S.F., Jiang Y.J., Zou J.W. (2015). Predicting retention and separation factors of chiral diarylmethane derivates by QSPR models. Acta Phys-Chim. Sin..

[90] Grecsó N., Kohout M., Carotti A., Sardella R., Natalini B., Fülöp F., Lindner W., Péter A., Ilisz I. (2016). Mechanistic considerations of enantiorecognition on novel Cinchona alkaloid-based zwitterionic chiral stationary phases from the aspect of the separation of trans-paroxetine enantiomers as model compounds. J. Pharm. Biomed. Anal..

[91] Hu G., Huang M., Luo C., Wang Q., Zou J.W. (2016). Interactions between pyrazole derived enantiomers and Chiralcel OJ: Prediction of enantiomer absolute configurations and elution order by molecular dynamics simulations. J. Mol. Graph. Mod..

[92] Peluso P., Mamane V., Aubert E., Dessì A., Dallocchio R., Dore A., Pale P., Cossu S. (2016). Insights into halogen bond-driven enantioseparations. J. Chromatogr. A.

[93] Szabó Z.I., Mohammadhassan F., Szőcs L., Nagy J., Komjáti B., Noszál B., Tóth G. (2016). Stereoselective interactions and liquid chromatographic enantioseparation of thalidomide on cyclodextrin-bonded stationary phases. J. Incl. Phenom. Macrocyl Chem..

[94] Szabó Z.I., Szőcs L., Horváth P., Komjáti B., Nagy J., Jánoska Á., Muntean D.L., Noszál B., Tóth G. (2016). Liquid chromatography with mass spectrometry enantioseparation of pomalidomide on cyclodextrin-bonded chiral stationary phases and the elucidation of the chiral recognition mechanisms by NMR spectroscopy and molecular modeling. J. Sep. Sci..

[95] Xie J., Zhao L., Liu K., Guo F., Liu W. (2016). Enantioseparation of four amide herbicide stereoisomers using high-performance liquid chromatography. J. Chromatogr. A.

[96] Çakmak R., Ercan S., Sünkür M., Yılmaz H., Topal G. (2017). Design, preparation and application of a Pirkle-type chiral stationary phase for enantioseparation of some racemic organic acids and molecular dynamics studies. Org. Commun..

[97] Carraro M.L., Palmeira A., Tiritan M.E., Fernandes C., Pinto M.M. (2017). Resolution, determination of enantiomeric purity and chiral recognition mechanism of new xanthone derivatives on (*S*,*S*)-whelk-O1 stationary phase. Chirality.

[98] Li X., Yao X., Xiao Y., Wang Y. (2017). Enantioseparation of single layer native cyclodextrin chiral stationary phases: Effect of cyclodextrin orientation and a modeling study. Anal. Chim. Acta.

[99] Rossi D., Nasti R., Collina S., Mazzeo G., Ghidinelli S., Longhi G., Memo M., Abbate S. (2017). The role of chirality in a set of key intermediates of pharmaceutical interest, 3-aryl-substituted-γ-butyrolactones, evidenced by chiral HPLC separation and by chiroptical spectroscopies. J. Pharm. Biomed. Anal..

[100] Zhao B., Oroskar P.A., Wang X., House D., Oroskar A., Oroskar A., Jameson C., Murad S. (2017). The composition of the mobile phase affects the dynamic chiral recognition of drug molecules by the chiral stationary phase. Langmuir.

[101] Dallocchio R., Dessì A., Solinas M., Arras A., Cossu S., Aubert E., Mamane V., Peluso P. (2018). Halogen bond in high-performance liquid chromatography enantioseparations: Description, features and modelling. J. Chromatogr. A.

[102] Li M., Zhang B., Yu J., Wang J., Guo X. (2018). Enantiomeric separation and simulation study of eight anticholinergic drugs on an immobilized polysaccharide-based chiral stationary phase by HPLC. New J. Chem..

[103] Peluso P., Gatti C., Dessì A., Dallocchio R., Weiss R., Aubert E., Pale P., Cossu S., Mamane V. (2018). Enantioseparation of fluorinated 3-arylthio-4,4’-bipyridines: Insights into chalcogen and π-hole bonds in high-performance liquid chromatography. J. Chromatogr. A.

[104] Peluso P., Mamane V., Dallocchio R., Dessì A., Villano R., Sanna D., Aubert E., Pale P., Cossu S. (2018). Polysaccharide-based chiral stationary phases as halogen bond acceptors: A novel strategy for detection of stereoselective σ-hole bonds in solution. J. Sep. Sci..

[105] Pisani L., Rullo M., Catto M., de Candia M., Carrieri A., Cellamare S., Altomare C.D. (2018). Structure–property relationship study of the HPLC enantioselective retention of neuroprotective 7-[(1-alkylpiperidin-3-yl)methoxy]coumarin derivatives on an amylose-based chiral stationary phase. J. Sep. Sci..

[106] Sardella R., Macchiarulo A., Urbinati F., Ianni F., Carotti A., Kohout M., Lindner W., Péter A., Ilisz I. (2018). Exploring the enantiorecognition mechanism of Cinchona alkaloid-based zwitterionic chiral stationary phases and the basic trans-paroxetine enantiomers. J. Sep. Sci..

[107] Xiong F., Yang B.B., Zhang J., Li L. (2018). Enantioseparation, stereochemical assignment and chiral recognition mechanism of sulfoxide-containing drugs. Molecules.

[108] Zhu B., Zhao F., Yu J., Wang Z., Song Y., Li Q. (2018). Chiral separation and a molecular modeling study of eight azole antifungals on the cellulose tris(3,5-dichlorophenylcarbamate) chiral stationary phase. New J. Chem..

[109] Barfeii H., Garkani-Nejad Z., Saheb V. (2019). Investigation of the mechanism of enantioseparation of some drug compounds by considering the mobile phase in HPLC by molecular dynamics simulation. J. Mol. Model..

[110] Do Carmo J.P., Phyo Y.Z., Palmeira A., Tiritan M.E., Afonso C., Kijjoa A., Pinto M.M., Fernandes C. (2019). Enantioseparation, recognition mechanisms and binding of xanthones on human serum albumin by liquid chromatography. Bioanalysis.

[111] He Z., Wu F., Xia W., Li L., Hu K., Kaziem A.E., Wang M. (2019). Separation and detection of cyproconazole enantiomers and its stereospecific recognition with chiral stationary phase by high-performance liquid chromatography. Analyst.

[112] Knežević A., Novak J., Vinković V. (2019). New brush-type chiral stationary phases for enantioseparation of pharmaceutical drugs. Molecules.

[113] Li J., Liu R., Wang L., Liu X., Gao H. (2019). Enantioseparation of chiral pharmaceuticals by vancomycin-bonded stationary phase and analysis of chiral recognition mechanism. Chirality.

[114] Luo X., Fang C., Mi J., Xu J., Lin H. (2019). Enantiomeric resolution, thermodynamic parameters, and modeling of clausenamidone and neoclausenamidone on polysaccharide-based chiral stationary phases. Chirality.

[115] Phyo Y.Z., Teixeira J., Tiritan M.E., Cravo S., Palmeira A., Gales L., Silva A.M., Pinto M.M., Kijjoa A., Fernandes C. (2020). New chiral stationary phases for liquid chromatography based on small molecules: Development, enantioresolution evaluation and chiral recognition mechanisms. Chirality.

[116] Wu Q., Gao J., Chen L., Dong S., Li H., Qiu H., Zhao L. (2019). Graphene quantum dots functionalized β-cyclodextrin and cellulose chiral stationary phases with enhanced enantioseparation performance. J. Chromatogr. A.

[117] Zhao Y., Li S., Wang X., Yu J., Song Y., Guo X. (2019). Enantioseparation and molecular modeling study of five β-adrenergic blockers on C hiralpak IC column. Chirality.

[118] Bi W., Wang F., Han J., Liu B., Shen J., Zhang L., Okamoto Y. (2020). Influence of the substituents on phenyl groups on enantioseparation property of amylose phenylcarbamates. Carbohydr. Polym..

[119] Cai L., Xue M., Lun J., Li S., Yu J., Guo X. (2020). Enantioseparation and molecular modeling study of eight psychoactive drugs on a coated polysaccharide-based chiral stationary phase. Electrophoresis.

[120] Ianni F., Cerra B., Shandiz S.T., Di Michele A., Saluti G., Galarini R., Gioiello A., Sardella R., Carotti A. (2020). Integrating experimental and computational techniques to study chromatographic enantioresolutions of chiral tetrahydroindazole derivatives. J. Chromatogr. A.

[121] Li M., Jiang Z., Di X., Song Y. (2020). Enantiomeric separation of six beta-adrenergic blockers on Chiralpak IB column and identification of chiral recognition mechanisms by molecular docking technique. Biomed. Chromatogr..

[122] Liu Y., Cai L., Lun J., Zhao M., Guo X. (2020). Enantiomeric separation and molecular docking study of seven imidazole antifungal drugs on a cellulose tris-(3,5-dimethylphenylcarbamate) chiral stationary phase. New J. Chem..

[123] Papp L.A., Foroughbakhshfasaei M., Fiser B., Horváth P., Kiss E., Sekkoum K., Gyéresi Á., Hancu G., Noszál B., Szabó Z.I. (2020). Reversed-phase HPLC enantioseparation of pantoprazole using a teicoplanin aglycone stationary phase—Determination of the enantiomer elution order using HPLC-CD analyses. Chirality.

[124] Sardella R., Ianni F., Cossignani L., Aldini G., Carotti A. (2020). Binding modes identification through molecular dynamic simulations: A case study with carnosine enantiomers and the Teicoplanin A2-2-based chiral stationary phase. J. Sep. Sci..

[125] Shahnani M., Sefidbakht Y., Maghari S., Mehdi A., Rezadoost H., Ghassempour A. (2020). Enantioseparation of mandelic acid on vancomycin column: Experimental and docking study. Chirality.

[126] Shi G., Dai X., Zhou Y., Zhang J., Shen J., Wan X. (2020). Synthesis and enantioseparation of proline-derived helical polyacetylenes as chiral stationary phases for HPLC. Polym. Chem..

[127] Wang X., Jameson C.J., Murad S. (2020). Modeling enantiomeric separations as an interfacial process using amylose tris(3,5-dimethylphenyl carbamate)(ADMPC) polymers coated on amorphous silica. Langmuir.

[128] Yang Y., Hu J., Fang H., Hou X., Hou Z., Sang L., Yang X. (2020). Enantioseparation of lysine derivatives on amylose tris(3,5-dimethylphenylcarbamate) as chiral stationary phase with high separation factor. J. Chromatogr. A.

[129] Dallocchio R., Sechi B., Dessì A., Chankvetadze B., Cossu S., Mamane V., Weiss R., Pale P., Peluso P. (2021). Enantioseparations of polyhalogenated 4, 4’-bipyridines on polysaccharide-based chiral stationary phases and molecular dynamics simulations of selector–selectand interactions. Electrophoresis.

[130] Franzini R., Pierini M., Mazzanti A., Iazzetti A., Ciogli A., Villani C. (2020). Molecular recognition of the HPLC Whelk-O1 selector towards the conformational enantiomers of nevirapine and oxcarbazepine. Int. J. Mol. Sci..

[131] Haginaka J., Yamashita T., Tsujino H., Arisawa M. (2021). Revisiting Chiral Recognition Mechanism on Chicken Alpha 1-Acid Glycoprotein: Location of Chiral Binding Sites and Insight into Chiral Binding Mechanism. Separations.

[132] Hoyas S., Roscioni O.M., Tonneaux C., Gerbaux P., Cornil J., Muccioli L. (2021). Peptoids as a chiral stationary phase for liquid chromatography: Insights from molecular dynamics simulations. Biomacromolecules.

[133] Li M., Guo X., Di X., Jiang Z. (2021). Enantioseparation on a new synthetic β-cyclodextrin chemically bonded chiral stationary phase and molecular docking study. Anal. Bioanal. Chem..

[134] Li M., Jiang Z., Guo X., Di X., Yu J. (2021). Enantioseparation and modelling study of six proton pump inhibitors on a novel 3,5-dichloro-phenylcarbamated β-cyclodextrin chemically bonded chiral stationary phase by high performance liquid chromatography. Microchem. J..

[135] Liu Y., Wang X., Yu J., Guo X. (2021). Chiral separation and molecular simulation study of six antihistamine agents on a coated cellulose tris-(3,5-dimethylphenycarbamate) column (Chiralcel OD-RH) and its recognition mechanisms. Electrophoresis.

[136] Peluso P., Chankvetadze B. (2021). The molecular bases of chiral recognition in 2-(benzylsulfinyl) benzamide enantioseparation. Anal. Chim. Acta.

[137] Ratih R., Wätzig H., Azminah A., Asmari M., Peters B., El Deeb S. (2021). Immobilization of chondroitin sulfate a onto monolithic epoxy silica column as a new chiral stationary phase for high-performance liquid chromatographic enantioseparation. Pharm.

[138] Shi G., Dai X., Xu Q., Shen J., Wan X. (2021). Enantioseparation by high-performance liquid chromatography on proline-derived helical polyacetylenes. Polym. Chem..

[139] Varfaj I., Di Michele A., Ianni F., Saletti M., Anzini M., Barola C., Chankvetadze B., Sardella R., Carotti A. (2021). Enantioseparation of novel anti-inflammatory chiral sulfoxides with two cellulose dichlorophenylcarbamate-based chiral stationary phases and polar-organic mobile phase (s). J. Chromatogr. Open.

[140] Varfaj I., Protti M., Di Michele A., Macchioni A., Lindner W., Carotti A., Sardella R., Mercolini L. (2021). Efficient enantioresolution of aromatic α-hydroxy acids with Cinchona alkaloid-based zwitterionic stationary phases and volatile polar-ionic eluents. Anal. Chim. Acta.

[141] Wang X., Jameson C.J., Murad S. (2021). Molecular dynamics simulations of chiral recognition of drugs by amylose polymers coated on amorphous silica. Mol. Phys..

[142] Bolognino I., Carrieri A., Purgatorio R., Catto M., Caliandro R., Carrozzini B., Belviso B.D., Majellaro M., Sotelo E., Cellamare S. (2021). Enantiomeric Separation and Molecular Modelling of Bioactive 4-Aryl-3,4-dihydropyrimidin-2(1H)-one Ester Derivatives on Teicoplanin-Based Chiral Stationary Phase. Separations.

[143] Cao S., Ma Q., Liu Y., Zhang J., Wang Z. (2022). Cellulose tris-(3,5-dimethyl phenyl carbamate) as a chiral stationary phase for enantiomeric determination of ofloxacin enantiomers and molecular docking study on the chiral separation mechanism. New J. Chem..

[144] Cao S., Zhou Y., Ma Q., Zhang J., Wang Z. (2022). Experimental and computational studies of enantioseparation of three profen enantiomers with a focus on quantification of the enantiomeric impurities present in the corresponding enantiopure S-profen drugs. J. Chromatogr. A.

[145] Dallocchio R., Dessì A., Sechi B., Chankvetadze B., Cossu S., Mamane V., Aubert E., Rozzo C., Palmieri G., Spissu Y. (2022). Exploring interaction modes between polysaccharide-based selectors and biologically active 4, 4′-bipyridines by experimental and computational analysis. J. Chromatogr. Open.

[146] Deng H., Wu X., Zhang L., Shen J., Qiao Y., Wang X., Bai C., Zheng T., Okamoto Y. (2022). Synthesis and application of chitosan thiourea derivatives as chiral stationary phases in HPLC. Carbohydr. Polym..

[147] Dessì A., Sechi B., Dallocchio R., Chankvetadze B., Pérez-Baeza M., Cossu S., Mamane V., Pale P., Peluso P. (2022). Comparative enantioseparation of planar chiral ferrocenes on polysaccharide-based chiral stationary phases. Chirality.

[148] Gambacorta N., Özdemir Z., Doğan İ.S., Ciriaco F., Zenni Y.N., Karakurt A., Saraç S., Nicolotti O. (2022). Integrated experimental and theoretical approaches to investigate the molecular mechanisms of the enantioseparation of chiral anticonvulsant and antifungal compounds. J. Mol. Struct..

[149] Mousavimanesh Z., Shahnani M., Faraji-Shovey A., Bararjanian M., Sadr A.S., Ghassempour A., Salehi P. (2022). A new chiral stationary phase based on noscapine: Synthesis, enantioseparation, and docking study. Chirality.

[150] Samir L., Hanafi R., El Deeb S., Spahn-Langguth H. (2022). UHPLC Enantiomer Resolution for the α/β-Adrenoceptor Antagonist R/S-Carvedilol and Its Major Active Metabolites on Chiralpak IB N-5. Molecules.

[151] Shi G., Li Y., Dai X., Shen J., Wan X. (2022). Effect of pendant stereostructure on backbone conformation and enantioseparation ability of helical polyacetylene-based chiral stationary phases. Chirality.

[152] Mimouni F.Z., Belboukhari N., Sekkoum K., Aboul-Enein H.Y. (2022). Novel Gatifloxacin3-carboxamide derivatives as anti-tumor agents: Synthesis, enantioseparation, and molecular docking. Curr. Anal. Chem..

[153] Adhikari S., Bhujbal S., Paik M.J., Lee W. (2023). Enantioseparation and molecular modeling study of chiral amines as three naphthaldimine derivatives using amylose or cellulose trisphenylcarbamate chiral stationary phases. Chirality.

[154] Bai Q., Yu Y., Zhao P., Yang Y., Zhang Y., Tan C., Zhu Y., Fang L., Li L. (2023). Enantioselective separation and simulation studies of five flavanone glycosides on a cellulose tris-(3,5-dichlorophenylcarbamate) chiral stationary phase. J. Mol. Liq..

[155] Dallocchio R., Dessì A., Sechi B., Chankvetadze B., Jibuti G., Cossu S., Mamane V., Peluso P. (2023). Enantioseparation of planar chiral ferrocenes on cellulose-based chiral stationary phases: Benzoate versus carbamate pendant groups. Electrophoresis.

[156] Dobó M., Ádám M., Fiser B., Papp L.A., Dombi G., Sekkoum K., Szabó Z.I., Tóth G. (2023). Enantioseparation and molecular docking study of selected chiral pharmaceuticals on a commercialized phenylcarbamate-β-cyclodextrin column using polar organic mode. Sci. Rep..

[157] Dombi G., Horváth P., Fiser B., Mirzahosseine A., Dobó M., Szabó Z.I., Tóth G. (2023). Enantioselective Human Serum Albumin Binding of Apremilast: Liquid Chromatographic, Fluorescence and Molecular Docking Study. Int. J. Mol. Sci..

[158] Li Y., Jin X., Xiao Y., Ma X., Wang Y. (2023). Investigation of the chiral recognition role of cyclodextrin hydroxyl moieties via high performance liquid chromatography. Analyst.

[159] Li Y., Zhang Y., Lu X., Sun S., Xiao Y., Wang Y., Jin X., Ma X. (2023). Surface-up click access to allylimidazolium bridged cyclodextrin dimer phase for efficient enantioseparation. J. Sep. Sci..

[160] Nguyen B.T., Choi Y.J., Kim K.H., Song G.Y., Kim H.M., Kang J.S. (2023). Chiral separation and molecular modeling study of decursinol and its derivatives using polysaccharide-based chiral stationary phases. J. Chromatogr. A.

[161] Núñez-Rico J.L., Cabezas-Giménez J., Lillo V., Balestra S.R., Galán-Mascarós J.R., Calero S., Vidal-Ferran A. (2023). TAMOF-1 as a versatile and predictable chiral stationary phase for the resolution of racemic mixtures. ACS Appl. Mater. Interfaces.

[162] Protti M., Varfaj I., Carotti A., Tedesco D., Bartolini M., Favilli A., Gerli S., Mercolini L., Sardella R. (2023). Microsampling and enantioselective liquid chromatography coupled to mass spectrometry for chiral bioanalysis of novel psychoactive substances. Talanta.

[163] Saleh O.A., Badawey A.M., Aboul-Enein H.Y., Fouad M.A. (2023). Enantioseparation, quantification, molecular docking and molecular dynamics study of five β-adrenergic blockers on Lux-Cellulose-2 column. BMC Chem..

[164] Sechi B., Dessì A., Dallocchio R., Tsetskhladze N., Chankvetadze B., Pérez-Baeza M., Cossu S., Jibuti G., Mamane V., Peluso P. (2023). Unravelling dispersion forces in liquid-phase enantioseparation. Part I: Impact of ferrocenyl versus phenyl groups. Anal. Chim. Acta.

[165] Zhang C., Li W., Ning B. (2023). Enantiomeric Resolution of Pidotimod and Its Isomers in Pidotimod Oral Solutions Using Chiral RP-HPLC with Quadrupole Dalton Analyzer Detection. Chromatographia.

[166] Varfaj I., Labikova M., Sardella R., Hettegger H., Lindner W., Kohout M., Carotti A. (2024). A journey in unraveling the enantiorecognition mechanism of 3,5-dinitrobenzoyl-amino acids with two Cinchona alkaloid-based chiral stationary phases: The power of molecular dynamic simulations. Anal. Chim. Acta.

[167] Reshetova E.N., Barashkova A.S., Garifullin B.F. (2024). Retention mechanisms of dipeptides on superficially porous particle vancomycin-and teicoplanin-based chiral stationary phases. J. Chromatogr. A.

[168] Zhang C., Wang Y., Li Y., Song J., Wang Y. (2024). Click preparation of triazole-bridged teicoplanin-bound chiral stationary phases for efficient separating amino acid enantiomers. Talanta.

[169] Saleh O.A., Badawey A.M., Enein H.Y., Mahmoud S.T. (2024). An innovative combination of molecular modeling and green analysis approaches for the enantioseparation and quantitation of certain CNS acting drugs using HPLC. Microchem. J..

[170] Li T., Li H., Chen J., Yu Y., Chen S., Wang J., Qiu H. (2024). Preparation and evaluation of two chiral stationary phases based on imidazolyl-functionalized bromoethoxy pillar [5] arene-bonded silica. J. Chromatogr. A.

[171] Bonafè S., Pagano C., Bianconi E., Mercolini L., Macchiarulo A., Perioli L., Sardella R., Carotti A. (2024). Atypical enantioseparation of a non-ionic form of allantoin with Cinchona alkaloid-based zwitterionic chiral stationary phases. J. Chromatogr. Open.

[172] Coelho M.M., Lima R., Almeida A.S., Fernandes P.A., Remião F., Fernandes C., Tiritan M.E. (2024). Binding studies of promethazine and its metabolites with human serum albumin by high-performance affinity chromatography and molecular docking in the presence of codeine. Anal. Bioanal. Chem..

[173] Sri C.D., Faizan S., Chandra M.R., Kumar B.P., Gurupadayya B.M. (2024). Enantioselective Separation and Pharmacokinetics of a Chiral 1,4-Dihydropyrimidine Derivative in Rats: A Combined Chromatography and Docking Approach. Chirality.

[174] Bai H., Chen L. (2024). Stereoisomeric separation and chiral recognition mechanism study of star cyclodextrin polymer as the chiral stationary phase. Anal. Chim. Acta.

[175] Dombi G., Tyukodi L., Dobó M., Molnár G., Rozmer Z., Szabó Z.I., Fiser B., Tóth G. (2024). Enantioselective Binding of Proton Pump Inhibitors to Alpha1-Acid Glycoprotein and Human Serum Albumin—A Chromatographic, Spectroscopic, and In Silico Study. Int. J. Mol. Sci..

[176] Guarducci M.A., Manetto S., Pierini M., Mazzoccanti G., Villani C. (2024). Design, Synthesis, and Applications of Bis-Amido HPLC Pirkle-Type Chiral Stationary Phases. Chirality.

[177] Muhammed M.T., Aki-Yalcin E. (2024). Molecular docking: Principles, advances, and its applications in drug discovery. Lett. Drug Des. Discov..

[178] Jameson C.J., Wang X., Murad S. (2021). Molecular dynamics simulations of enantiomeric separations as an interfacial process in HPLC. AIChE J..

[179] Dascalu A.E., Speybrouck D., Billamboz M., Corens D., Ghinet A., Lipka E. (2020). Analytical and preparative enantioseparations in supercritical fluid chromatography using different brands of immobilized cellulose tris (3,5-dichlorophenylcarbamate) columns: Some differences. J. Chromatogr. A.

[180] Ali I., Saleem K., Hussain I., Gaitonde V.D., Aboul-Enein H.Y. (2009). Polysaccharides chiral stationary phases in liquid chromatography. Sep. Purif. Rev..

[181] Chen X., Yamamoto C., Okamoto Y. (2007). Polysaccharide derivatives as useful chiral stationary phases in high-performance liquid chromatography. Pure Appl. Chem..

[182] Ikai T., Ando M., Ito M., Ishidate R., Suzuki N., Maeda K., Yashima E. (2021). Emergence of highly enantioselective catalytic activity in a helical polymer mediated by deracemization of racemic pendants. J. Am. Chem. Soc..

[183] Cavazzini A., Pasti L., Massi A., Marchetti N., Dondi F. (2011). Recent applications in chiral high performance liquid chromatography: A review. Anal. Chim. Acta.

[184] Wang H., Shen J., Wu Y., Sun X., Ke Y. (2021). Enantioseparation of cloprostenol on the polysaccharide chiral stationary phase: Influence of the mobile phase on enantioselective adsorption. J. Chromatogr. A.

[185] Torres P.H., Sodero A.C., Jofily P., Silva-Jr F.P. (2019). Key topics in molecular docking for drug design. Int. J. Mol. Sci..

[186] Tripathi A., Misra K. (2017). Molecular docking: A structure-based drug designing approach. JSM Chem..

[187] Chaudhary K.K., Mishra N. (2016). A review on molecular docking: Novel tool for drug discovery. JSM Chem..

[188] Tao X., Huang Y., Wang C., Chen F., Yang L., Ling L., Che Z., Chen X. (2020). Recent developments in molecular docking technology applied in food science: A review. Int. J. Food Sci. Technol..

[189] Fan J., Fu A., Zhang L. (2019). Progress in molecular docking. Quant. Biol..

[190] Jamkhande P.G., Ghante M.H., Ajgunde B.R. (2017). Software based approaches for drug designing and development: A systematic review on commonly used software and its applications. Bull. Fac. Pharm..

[191] Wei B.Q., Baase W.A., Weaver L.H., Matthews B.W., Shoichet B.K. (2002). A model binding site for testing scoring functions in molecular docking. J. Mol. Biol..

[192] Li J., Fu A., Zhang L. (2019). An overview of scoring functions used for protein–ligand interactions in molecular docking. Interdiscip. Sci..

[193] Huang S.Y., Grinter S.Z., Zou X. (2010). Scoring functions and their evaluation methods for protein–ligand docking: Recent advances and future directions. Phys. Chem. Chem. Phys..

[194] Quiroga R., Villarreal M.A. (2016). Vinardo: A scoring function based on autodock vina improves scoring, docking, and virtual screening. PLoS ONE.

[195] Phyo Y.Z., Teixeira J., Goncalves R., Palmeira A., Tiritan M.E., Bousbaa H., Pinto M.M., Fernandes C., Kijjoa A. (2021). Chiral derivatives of xanthones and benzophenones: Synthesis, enantioseparation, molecular docking, and tumor cell growth inhibition studies. Chirality.

[196] Hollingsworth S.A., Dror R.O. (2018). Molecular dynamics simulation for all. Neuron.

[197] Zhao C., Cann N.M. (2007). The docking of chiral epoxides on the Whelk-O1 stationary phase: A molecular dynamics study. J. Chromatogr. A.

[198] Zhao C., Cann N.M. (2006). Solvation of the Whelk-O1 chiral stationary phase: A molecular dynamics study. J. Chromatogr. A.

[199] Nita S., Cann N.M. (2008). Solvation of phenylglycine-and leucine-derived chiral stationary phases: Molecular dynamics simulation study. J. Phys. Chem. B.

[200] Li Y., Liu D., Wang P., Zhou Z. (2010). Computational study of enantioseparation by amylose tris (3,5-dimethylphenylcarbamate)-based chiral stationary phase. J. Sep. Sci..

[201] Bueno-Perez R., Balestra S.R., Camblor M.A., Min J.G., Hong S.B., Merkling P.J., Calero S. (2018). Influence of Flexibility on the Separation of Chiral Isomers in STW-Type Zeolite. Chem. Eur. J..

[202] Asmari M., Wang X., Casado N., Piponski M., Kovalenko S., Logoyda L., Hanafi R.S., El Deeb S. (2021). Chiral monolithic silica-based HPLC columns for enantiomeric separation and determination: Functionalization of chiral selector and recognition of selector-selectand interaction. Molecules.

[203] Shi J.H., Lin Z.Y., Kou S.B., Wang B.L., Jiang S.L. (2021). Enantioseparation of mandelic acid and substituted derivatives by high-performance liquid chromatography with hydroxypropyl-β-cyclodextrin as chiral mobile additive and evaluation of inclusion complexes by molecular dynamics. Chirality.

[204] Joshi S.Y., Deshmukh S.A. (2021). A review of advancements in coarse-grained molecular dynamics simulations. Mol. Simul..

[205] Fedotov A., Vakhrushev A., Severyukhina O., Sidorenko A., Savva Y., Klenov N., Soloviev I. (2021). Theoretical basis of quantum-mechanical modeling of functional nanostructures. Symmetry.

[206] Lahoz-Beltra R. (2022). Solving the Schrödinger Equation with Genetic Algorithms: A Practical Approach. Computers.

[207] Chen E.K. (2019). Realism about the wave function. Philos. Compass.

[208] Car R. (2002). Introduction to Density-Functional Theory and ab-Initio Molecular Dynamics. Quant. Struct.-Act. Relatsh..

[209] Kumar A., Toal S.E., DiGuiseppi D., Schweitzer-Stenner R., Wong B.M. (2020). Water-mediated electronic structure of oligopeptides probed by their UV circular dichroism, absorption spectra, and time-dependent DFT calculations. J. Phys. Chem. B.

[210] Kumar A., Schweitzer-Stenner R., Wong B.M. (2019). A new interpretation of the structure and solvent dependence of the far UV circular dichroism spectrum of short oligopeptides. Chem. Comm..

[211] Miyahara T., Nakatsuji H. (2018). Accuracy of TD-DFT in the ultraviolet and circular dichroism spectra of deoxyguanosine and uridine. J. Phys. Chem. A.

